# Development and cross-species transferability of EST-SSR markers in Siberian wildrye (*Elymus sibiricus* L.) using Illumina sequencing

**DOI:** 10.1038/srep20549

**Published:** 2016-02-08

**Authors:** Qiang Zhou, Dong Luo, Lichao Ma, Wengang Xie, Yu Wang, Yanrong Wang, Zhipeng Liu

**Affiliations:** 1 The State Key Laboratory of Grassland Agro-ecosystems, College of Pastoral Agriculture Science and Technology, Lanzhou University, Lanzhou, 730020, China

## Abstract

Siberian wildrye (*Elymus sibiricus* L.) is a perennial, self-fertilizing grass that plays an important role in animal husbandry and environmental sustenance. However, the transcriptomic and genomic information on this species is very limited, which hinders genetic and breeding studies. In the present study, 76,686,804 clean reads were generated from 11 different tissue samples of *E. sibiricus* by Illumina paired-end sequencing, and the reads were deposited into the NCBI SRA database (SRX574376). A total of 8,769 EST-SSRs were identified from 94,458 unigene sequences, which were obtained by *de novo* assembly. Moreover, 1,078 primer pairs were successfully designed, and 500 pairs were randomly selected to assess polymorphisms in 15 *E. sibiricus* accessions. A total of 112 primer pairs were polymorphic, and the polymorphism information content (*PIC*) values ranged from 0.39 to 0.81, indicating a high level of informativeness. Furthermore, these 112 polymorphic primer pairs were used to evaluate the transferability to 13 other related species, and 55 EST-SSR markers were found to be polymorphic among these 13 *Elymus* species. This study collected the global sequence data for *E. sibiricus,* and the newly developed markers will prove valuable in facilitating genetic diversity in *E. sibiricus* and related *Elymus* species.

Siberian wildrye (*Elymus sibiricus* L.) is the typical species of the genus *Elymus*, which is the largest genus in the Triticeae family, and there are approximately 150 species of this genus distributed in most temperate regions of the world^1^. *E. sibiricus* is a perennial, self-fertilizing grass and an allotetraploid with the StStHH genome constitution (2*n* = 28)^2^. Moreover, *E. sibiricus* has the characteristics of good palatability, high yield, rich in nutrients, and high digestibility, which are conducive to the growth and reproduction of domestic animals. It has been widely used as an important forage grass in cultivated pastures and natural grassland, particularly in the Qinghai-Tibet plateau, due to its excellent cold and drought tolerance, high forage quality, good adaptability to the local environment and important role in animal husbandry and environmental sustenance^3^. However, recent research has suggested that climate warming and excessive grazing threaten the productivity and growth of *E. sibiricus*^4^. Therefore, it is important to study its conservation and exploitation for germplasm, and knowledge of the genetic diversity and population genetic structure of a species is a prerequisite for the successful management of conservation programs^5^. However, the transcriptomic and genomic information of *E. sibiricus* is very limited, hindering its use in genetic and breeding studies.

Simple sequence repeats (SSRs) or microsatellite markers are tandem repeated sequences comprising mono-, di-, tri-, tetra-, penta- or hexa-nucleotide units that possess high information content, co-dominance, and locus specificity and are easy to detect compared with other molecular markers^6^. SSRs have been used as a powerful tool in studies of genetic variation, genetic mapping, and molecular breeding^7^,^8^,^9^,^10^,^11^. Compared with genomic-SSRs, EST-SSRs have a higher level of transferability across related species because EST-SSRs originate from the transcribed regions in genomes and possess conserved sequences among homologous genes^12^.

To date, many SSRs have been developed in many plants through Illumina sequencing; these plants include alfalfa^13^,^14^, *Vicia sativa*^15^, and Indian sesame^16^. However, in the genus *Elymus*, the applications of SSRs have only been presented in a few species, including *E. alaskanus*^5^, *E. caninus*^17^, *E. trachycaulus*^18^, and *E*. *sibiricus*^19^. Most of these reported SSR primers were derived from barley and wheat microsatellite markers until a recent study reported that 53 genomic-SSRs were developed in *E*. *sibiricus*^4^. These novel SSR markers are the first characterized in *E. sibiricus* and will be useful for investigating genetic diversity and molecular-assisted breeding. However, these SSRs are still insufficient for genetic applications compared with those in some other plants, and there are 1,281 polymorphic EST-SSRs in peanut^20^.

Transcriptome sequencing is an efficient method to generate genomic-level data, large EST sequences, and molecular markers^21^. In recent years, next-generation sequencing technology has emerged as a cutting-edge approach for high-throughput sequence determination^22^. Additionally, it not only allows rapid and comprehensive analyses of the plant genome but also offers a cost-effective means of analysing gene transcripts^23^. Next-generation sequencing has been successfully and increasingly used in most plants, such as rice^24^, alfalfa^13^,^25^, *V. sativa*^26^, and barley^27^, but it has not yet been applied to research on *E*. *sibiricus* or even other species belonging to the *Elymus* genus.

The present study involves the first transcriptome sequencing of 11 *E*. *sibiricus* tissues using the Illumina Hiseq2000 sequencing platform. The objective of this study was to achieve a valuable sequence resource and develop some high polymorphism EST-SSR markers that would allow a better understanding of the genetic diversity in both *E. sibiricus* and the *Elymus* genus, which may be useful in modern *E. sibiricus* breeding programs.

## Results

### Sequencing and *de novo* assembly

**Figure 1:**
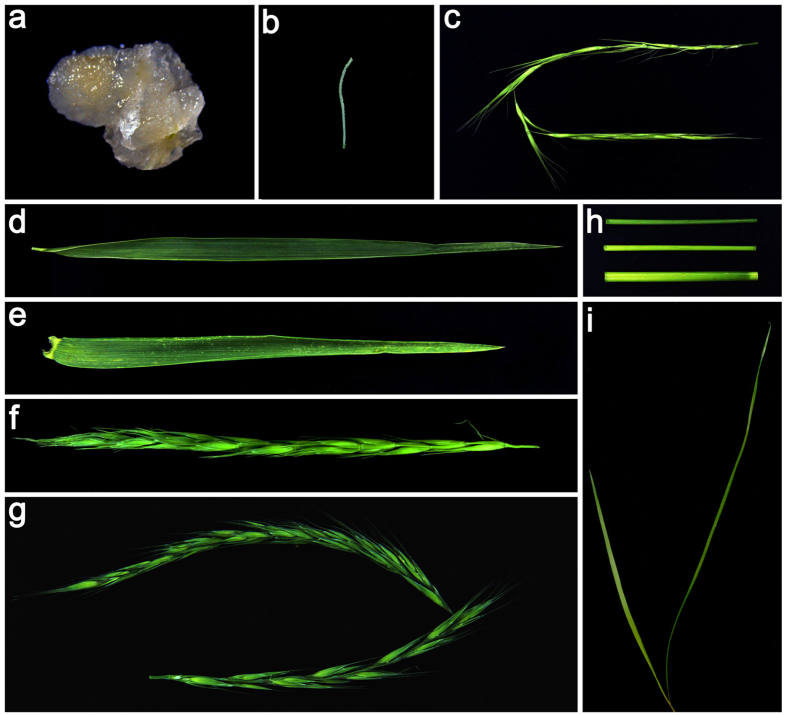
Representative tissues and samples used in this study. (**a**) Callus cells (induced by young inflorescences). (**b**) Radicles (seven days after seed germination). (**c**) Young inflorescences (10 days before fertilization). (**d**) Tufted leaves in the tillering stage. (**e**) Flag leaves in the heading stage. (**f**) Inflorescences (five days before fertilization). (**g**) Old inflorescences (five days after fertilization). (**h**) Stems (less lignified stems, moderately lignified stems and highly lignified stems). (**i**) Whole seedlings (three weeks after seed germination).

The constructed cDNA library from 11 distinct tissues (Fig. 1) was sequenced and generated 84,905,976 raw reads, which contained the adapter-primer sequences, low-quality sequences, and empty reads (Table 1). After a rigorous quality check and data filtering, a total of 76,686,804 high-quality clean reads with 97.97% Q20 bases were obtained. The clean reads had a total nucleotide number of 6,901,812,360 nt, and the N and GC percentages for the clean reads were 0 and 54.70%, respectively. Additionally, the high-quality reads were deposited into the U.S. National Center for Biotechnology Information (NCBI) sequence read archive (SRA) database (SRX574376).

As a result, 246,164 contigs with a mean length of 268 bp and an N50 length of 356 bp were obtained after *de novo* assembly. The total number of unigenes with paired-end reads was 94,458, including 42,058 distinct clusters and 52,400 distinct singletons, and the total length of the unigenes was 60,972,579 bp, with an average length of 645 bp and an N50 value of 942 bp. Among the 94,458 unigenes, the length of 76,500 unigenes (80.99%) ranged from 200 to 1,000 bp, the length of 17,385 unigenes (18.41%) ranged from 1,000 to 3,000 bp, and 573 unigenes (0.61%) were more than 3,000 bp in length. The length distributions of the unigenes are shown in Fig. S1.

Of the 94,458 unigenes, 75,384 (79.81%) unigenes were successfully annotated in the Nr, Nt, Swiss-Prot, KEGG, COG, and GO databases (Table 2), and 21,406 (22.66%) unigenes were assigned to the COG classifications (Fig. S2). After searching all 94,458 unigenes against the Nr database, 41,711 unigenes were assigned to one or more GO terms based on 62,046 Nr annotations, and these terms could be grouped into the following three main categories: biological process, cellular component, and molecular function (Fig. S3).

### Frequency and distribution of SSRs

A total of 8,769 potential EST-SSRs were identified from 7,732 unigenes (Table 1), and 1,078 primer pairs were successfully designed. Of these unigenes, 902 unigenes contained more than one EST-SSR. An average of one EST-SSR was found every 6.95 kb, and the frequency of SSRs was 8.19%. The type and distribution of the 8,769 potential EST-SSRs were then investigated. The most abundant type of repeat was tri-nucleotide repeats (5,319, 60.66%), followed by di-nucleotide (2,086, 23.79%), mono-nucleotide (444, 5.06%), penta-nucleotide (426, 4.86%), quad-nucleotide (303, 3.46%), and hexa-nucleotide (191, 2.18%) repeats (Fig. S4). As shown in Table 3, EST-SSRs with five tandem repeats (43.43%) were the most common, and these were followed by six tandem repeats (25.54%), seven tandem repeats (10.38%), and four tandem repeats (6.52%), whereas the remaining tandem repeats each accounted for less than 5% of the EST-SSRs. The EST-SSR length ranged from 12 to 24 bp, and 15 bp was the most frequently observed length (36.53%). Furthermore, a total of 317 motif sequence types were identified, including 25, 24, 40, 45, 90, and 93 types of mon-, di-, tri-, tetra-, penta-, and hexa-nucleotide repeats, respectively. The most dominant dinucleotide repeat was AG/CT (1,264, 60.59%), and CCG/CGG was the most abundant trinucleotide repeat motif, accounting for 33.84% of these repeats, and the most common repeat motif in all EST-SSRs (1,800, 20.53%) (Fig. S4, Table S1). Additionally, the GO enrichment of these 7,732 SSR-containing unigenes was executed using agriGO (http://bioinfo.cau.edu.cn/agriGO/)^28^ with the annotations of the assembled 94,458 unigenes as a reference. As a result, the proportion of “transcription” (GO: 0006350)-related unigenes was significantly increased, indicating that “transcription”-related unigenes were significantly enriched (Fig. S5).

### Development of EST-SSR markers

**Figure 2:**
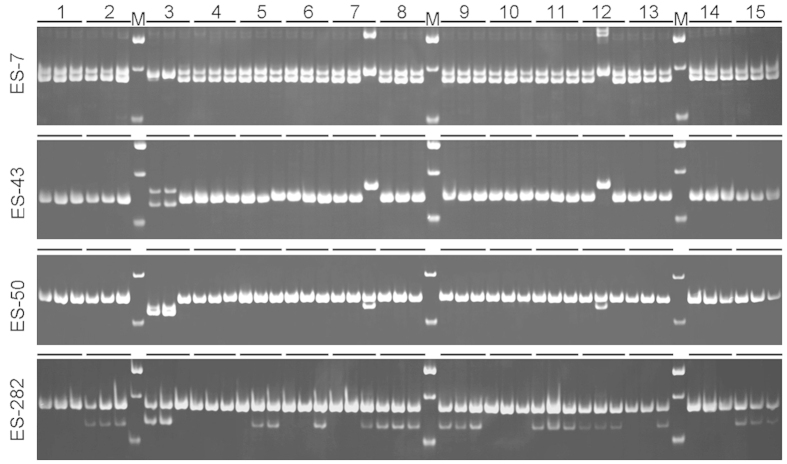
>EST-SSR marker variations at the ES-7, ES-43, ES-50, and ES-282 loci of 15 *E. sibiricus* accessions. Each accession includes three individual plants; the letter ‘M’ denotes the molecular markers, which are 200 bp, 150 bp, and 100 bp (top to bottom) in ES-7, ES-43, and ES-282 and 150 bp and 100 bp in ES-50 (top to bottom).

Based on the SSR-containing sequences, 500 of 1,078 primer pairs were randomly selected and synthesized to investigate whether the potential EST-SSR loci that were mined were true-to-type ones for use in population genetics. Among the 500 primer pairs selected, 438 successfully amplified the genomic DNA of *E*. *sibiricus* (Table S2), and the remaining 62 pair primers failed to amplify the PCR products at various annealing temperatures. Of the 438 successful primer pairs, 369 were able to yield amplification products of the expected size, and the other 69 primer pairs generated PCR products that were larger or smaller than expected. Using 45 *E*. *sibiricus* individuals from 15 accessions as PCR templates (Table S3), 112 of the 369 primer pairs were found to be polymorphic (Fig. 2, Table S4), whereas 257 were identified as monomorphic.

**Figure 3:**
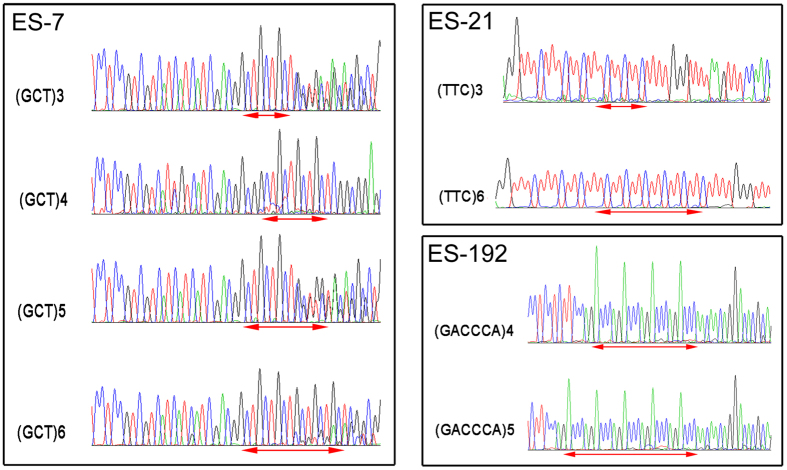
Comparative electropherogram analysis of three EST-SSR loci (ES-7, ES-21, and ES-192) among different populations of *E. sibiricus*.

In total, 553 alleles were detected at the 112 polymorphic loci in the 45 genotyped individuals, and the number of alleles ranged from three to nine with an average of 4.94. Estimates of the observed heterozygosity (*Ho*), expected heterozygosity (*He*), and polymorphism information content (*PIC*) ranged from 0 to 1, 0.51 to 0.83, and 0.39 to 0.81, with mean values of 0.49, 0.59, and 0.50, respectively. Detailed information for the 112 polymorphic primer pairs is shown in Table S4. Furthermore, PCR amplicons of three EST-SSRs from different individuals were sequenced to check the authenticity of the SSR locus. In all of the cases, the sequenced alleles from the different individuals were homologous to the original locus from which the marker was designed (Fig. 3). Using the unweighted pair-group method with arithmetic mean (UPGMA) and FreeTree program^29^, 15 germplasms of *E*. *sibiricus* were clustered into clusters A and B supported by bootstrap values of 0.97 and 1.00, respectively (Fig. S6). Cluster A contained 14 germplasms, whereas cluster B comprised only one germplasm, Pop3, which originated from Luqv, Gansu Province, China. Moreover, cluster A was divided into two groups, clusters C and D. The accessions in cluster C originated from Lintan, Zhuoni, Xiahe, and Maqv, Gansu Province and Ruoergai, Sichuan Province, whereas those in cluster D originated from Hezuo and Xiahe, Gansu Province, indicating that there is no clear relationship between the clustering pattern and geographical distance.

### Transferability of the newly developed EST-SSR markers

As a result, 55 out of the 112 primer pairs successfully amplified all of the accessions and displayed high polymorphism (Fig. S7, Table S4). A total of 327 alleles were discovered at the 55 polymorphic loci in the 41 genotyped individuals. To evaluate the polymorphic information of 55 loci within a species, the number of alleles, *Ho*, *He*, and *PIC* were calculated within each 13 different *Elymus* species (Table S5). The number of alleles ranged from 1.62 to 2.91 with an average of 2.16, and the mean values of *Ho*, *He*, and *PIC* were 0.56, 0.40, and 0.34, respectively. In addition, the PCR amplicons of two developed EST-SSRs from different species were sequenced to assess cross-species conservation and transferability. These sequence files were analysed, and the results unequivocally confirmed cross-species conservation and transferability but did not reflect significant differences (Fig. S8). The UPGMA tree revealed that 13 species were grouped into four clusters supported by bootstrap values ranging from 0.55 to 1.00 (Fig. S9). Cluster A comprised nine species, namely *E. abolinii*, *E. gmelinii*, *E. antiquus*, *E. ciliaris*, *E. tschimganicus*, *E. burchan-buddae*, *E. semicostatus*, *E. barbicallus*, and *E. macrochaetus*. In contrast, cluster B comprised *E. caninus* and *E. nevskii*, whereas clusters C and D comprised only one species, namely *E. longearistatus* and *E. panormitanus*, respectively.

## Discussion

Traditional Sanger sequencing technology can not meet the developmental needs emerging due to the progress of large-scale genomics^30^, whereas next-generation sequencing overcame the current limitations of Sanger sequencing with respect to throughput and costs. Next-generation sequencing has been widely used to analyse transcriptome sequencing and assembly in many plants because of its high efficiency, speed, accuracy, and low cost. However, next-generation sequencing has not been applied to research on *E*. *sibiricus.* In the present study, we used the Illumina HiSeq^TM^ 2000 platform to profile the *E*. *sibiricus* transcriptome from 11 distinct tissues, and a total of 76.69 million clean reads with a length of 6,901,812,360 bp were generated. In addition, 97.97% of the clean reads had Phred quality scores at the Q20 level and an N percentage (percentage of ambiguous “N” bases) of 0, which ensure the quality of the sequencing and is consistent with the results reported in *Dysosma versipellis*^31^. Next, 94,458 unigenes were assembled from the *E*. *sibiricus* transcriptome with a mean unigene length of 645 bp. This length was longer than that reported in other studies, involving, for example, tea (402 bp)^32^ and sweet potato (581 bp)^33^, possibly because the paired-end reads (100 bp) obtained in this study were longer than those obtained in previous studies (75 bp)^21^. However, the length was shorter than that documented in other reports, such as those describing studies in alfalfa (803 bp)^13^ and seashore paspalum (970 bp)^21^. This result may be due to the fact that the percentage of long sequences (more than 1,000 bp) in the *E*. *sibiricus* transcriptome (19.01%) was smaller than that calculated in alfalfa (26.97%) and seashore paspalum (35.48%). Moreover, it may be related to the difference in the assembler and the parameters as well as the nature of the species. For example, a longer mean length of unigenes in alfalfa (*Medicago sativa*) can be explained by the well-assembled reference genome of *M. truncatula*.

The assembled unigenes were subjected to BLAST analysis against the known databases, and a total of 75,384 (79.81%) unigenes were annotated. Additionally, 65.69% of the unigenes were identified by searching with BLASTX against the Nr database, and this percentage is higher than that obtained for other plants, such as orchid (49.25%)^34^, sesame (53.91%)^35^, wax gourd (55.4%)^36^, and litchi (59.65%)^37^. Furthermore, limited genomic and transcriptomic information is currently available for *E*. *sibiricus*, influencing the annotation efficiency, and some unigenes without BLAST hits may function as specific *E*. *sibiricus* genes.

In the present research, a total of 8,769 potential EST-SSRs were identified in 7,732 unigenes, and the frequency of the occurrence of EST-SSRs was one SSR in every 6.59 kb, which is much higher than those obtained for tree peony (1/9.24 kb)^12^, alfalfa (1/12.06 kb)^13^, pineapple (1/13 kb)^38^, and lotus (1/13.04 kb)^39^. However, this frequency is lower than those obtained in Levant cotton (1/2.4 kb)^40^, castor bean (1/1.77 kb)^41^, radish (1/3.45 kb)^42^, and gerbera (1/5.6 kb)^43^. It has been speculated that the frequency of SSRs strongly depends on the size of the databases, SSR search criteria, and mining tools used^44^,^45^. In this study, trinucleotide repeats were the most abundant type, which is consistent with the results obtained in presented studies on alfalfa^13^, tea^32^, and radish^42^. As shown in Fig. S4, the most dominant trinucleotide repeat motif was CCG/CGG, and the same result was found in seashore paspalum^21^, but AAG/CTT was the most abundant type in rubber tree^46^ and sesame^35^, indicating that the EST-SSR abundance usually differs between species. Among the dinucleotide repeats, AG/CT was the most frequent motif in our dataset, which is similar to that found in sesame^35^, radish^47^, and sweet potato^33^. One possible explanation is that CT motifs frequently occur in 5’ UTRs and may play an important role in gene regulation^21^,^35^. Furthermore, the results of the GO enrichment analysis showed that unigenes related to the category “transcription” were significantly enriched. Similar GO analyses of the SSR-containing unigenes in our published data for alfalfa^13^ and *V. sativa*^26^ were performed, and the results revealed that “transcription”-related unigenes were also significantly enriched, indicating that “transcription”-related unigenes may be more likely to contain SSR repeats than other unigenes^48^.

Of the 500 pair primers that were randomly selected for PCR validation, 438 (87.60%) produced clear bands. This PCR success rate was higher than the rates reported for alfalfa (30%)^49^, tree peony (47.30%)^12^, and rubber tree (59.8%)^50^. Among the successful primer pairs, 369 amplified PCR products were of the expected sizes, and 69 primer pairs resulted in larger or smaller PCR products than expected. These deviations may be attributed to the presence of introns, large insertions or repeat number variations, a lack of specificity, or assembly errors^33^,^35^. Nonetheless, 112 of those 369 primer pairs were polymorphic among 45 individuals of *E*. *sibiricus*; thus, the percentage of polymorphic loci in the tested species was 30.35%, which is higher than the results reported by Lei *et al.* (16.06%)^4^ but lower than that obtained in some of previous studies^46^,^51^,^52^. The decreased levels of polymorphism may be due to the smaller number or close geographic origin of the materials used in the study^12^. In the present study, 112 EST-SSR variations were found in the coding regions, whereas five were found in genes not associated with known proteins, which is similar to the location of EST-SSR markers in common vetch^15^.

In addition, the number of alleles for polymorphic markers ranged from three to nine with a mean of 4.94, and these values are higher than those reported by Lei *et al.*^4^ which ranged from two to five with an average of 3.09. These results indicate that the EST-SSR markers developed in this study had a higher level of polymorphism compared with the genomic SSR markers reported by Lei *et al.*^4^. Furthermore, a series of achievements on the genetic diversity of wild *E. sibiricus* germplasm and populations were reported^3^,^19^,^53^,^54^, and these showed a clear demarcation between accessions from different regions. However, the dendrogram of 15 *E*. *sibiricus* accessions obtained in the present study did not show any clear geographical patterns, which may be due to the lack of adequate accession numbers and the fact that these *E*. *sibiricus* accessions were sampled from adjacent areas, where the frequent exchange of *E*. *sibiricus* germplasm may obscure an existing pattern following the geographical origin of the accessions. Therefore, the use of a higher number of accessions from close geographical locations and more individual plants per accession will be essential for verifying the genetic diversity of *E*. *sibiricus* in future studies^14^.

In this research, we applied 112 newly developed EST-SSR markers to 13 species of the *Elymus* genus to evaluate the transferability of these EST-SSR markers as well as to offer some polymorphic EST-SSR markers to 13 other species. In total, 55 of the 112 primer pairs successfully amplified all of the species and obtained moderate transferability (49.11%), which is similar to that reported by Lei *et al.*^4^ and higher than that obtained in bottle gourd (4 to 41%)^55^ and *Cucumis* (12.7%)^56^. This moderate transferability of EST-SSRs in *E*. *sibiricus* was partly due to the moderate conservation of the sequences flanking the SSR among these 13 related species. The average of *PIC* ranged from 0.21 for *E. caninus* to 0.47 for *E. nevskii* at the 55 newly developed loci. Although the average of *PIC* for *E. nevskii* (0.47) and *E. panormitanus* (0.46) were similar to *E. sibiricus* (0.48), the values of other 11 species were less than *E. sibiricus* (Fig. S5). The reason could be related with 45 individual plants were selected in *E. sibiricus* while only three individual plants were selected in other 13 *Elymus* species. The EST-SSR markers that were developed from *E*. *sibiricus* offer a feasible solution for both correlational research of other related species that lack molecular markers and the study of comparative genomics in the *Elymus* genus. As shown in the dendrogram, *E. abolinii*, *E. ciliaris*, *E. tschimganicus*, and *E. burchan-buddae* were clustered into the same groups or subgroups, which is consistent with the findings obtained in previous studies^57^,^58^. However, part of our clustering results differ from those reported in previous studies of *E. abolinii* and *E. nevskii*^57^,^59^, suggesting that the use of a greater number of EST-SSR loci and a greater number of individuals per species would be essential to verify the relationship among *Elymus* species in future studies.

## Conclusions

To the best of our knowledge, this study describes the first assembly and characterization of the transcriptome of *E*. *sibiricus* using the Illumina paired-end sequencing method. This work presents a *de novo* transcriptome sequencing analysis of mixed RNAs from 11 different tissues. A total of 94,458 unigenes were generated, and 8,769 EST-SSRs were identified, providing a solid foundation for molecular marker development in *E*. *sibiricus*. Of these EST-SSRs, 1,078 primer pairs were successfully designed, and 500 were randomly selected for further validation. A total of 112 polymorphic primer pairs successfully amplified fragments, revealing abundant polymorphisms between 15 *E*. *sibiricus* accessions. Additionally, of these 112 polymorphic primer pairs, 55 were transferable among 13 other *Elymus* species, indicating that these 55 newly developed primer pairs can be used with confidence in future population genetic studies of the 13 related species. This study provides a valuable sequence resource for novel gene discovery and analysis of the genetic diversity in both *E*. *sibiricus* and the *Elymus* genus.

## Methods

### Tissue material and RNA isolation

In this study, a total of 11 tissue samples from *E*. *sibiricus* were collected, including callus cells (induced by young inflorescences), radicles (seven days after seed germination), whole seedlings (three weeks after seed germination), tufted leaves in the tillering stage, flag leaves in the heading stage, less lignified stems, moderately lignified stems, highly lignified stems, young inflorescences (10 days before fertilization), inflorescences (five days before fertilization), and old inflorescences (five days after fertilization) (Fig. 1). The callus cells were induced from young spikes on solid MS medium containing 2,4-dichlorophenoxyacetic acid (3.0 mg/L) at 25 °C for 30 d under 16-h-light/8-h-dark cycles. In addition, radicles and whole seedlings were obtained through seed germination and from different individual plants, but other tissues were collected from the same plant that grew for two years. The plants of *E*. *sibiricus* were grown in a greenhouse under a 16-h-light/8-h-dark cycle at 22 °C at Lanzhou University, Lanzhou, China. All of the sampled tissues were immediately placed in liquid nitrogen and stored at −80 °C until RNA extraction. The total RNA from 11 samples was isolated using the RNeasy Plant Mini Kit (Qiagen, Cat. #74904) according to the manufacturer’s instructions. The concentration of each sample must be greater than 600 ng/μl for transcriptome sequencing, as was determined using a NanoDrop ND1000 spectrophotometer (Thermo Scientific, USA).

### cDNA library construction and sequencing

To cover more tissue-specific transcripts in *E. sibiricus*, every sample was adjusted to the same concentration (400 ng/μl), and a total of 20 μg of RNA was pooled equally from the 11 tissues for preparation of the cDNA library. The cDNA library construction was conducted via the mRNA-Seq Sample Preparation Kit (Illumina Inc.) according to the manufacturer’s instructions. Briefly, the poly (A) mRNA was isolated by magnetic oligo (dT) beads, and first-strand cDNA was detected using random hexamer primers and reverse transcriptase (Invitrogen). The short cDNA fragments were then purified using a MinElute PCR Purification Kit (Qiagen) and resolved with EB buffer (Qiagen) for end reparation and adding poly (A). Finally, sequencing adapters were ligated to the fragments. The libraries were sequenced using the Illumina HiSeq2000 sequencing platform at the BGI TECH Company (Shenzhen, China). In addition, the processing of the fluorescent images for sequence base-calling and calculation of quality values was performed using the Illumina data processing pipeline, which yielded 100 bp paired-end reads.

### Sequence assembly and annotation

All of the raw reads were filtered before assembly, and this filtering included the removal of poly (A/T), low-quality sequences, and empty reads or reads with more than 10% of bases having Q < 20. The *de novo* transcriptome assembly of these clean reads was performed using the short read assembling program Trinity^21^. Contigs are longer fragments lacking N that were obtained by combined reads with a certain degree of overlap. Paired-end reads were used to obtain reads that were mapped back to contigs, which allows the detection of contigs from the same transcript as well as the distances between these contigs. Scaffolds were then produced via N, which represents unknown sequences between each set of two contigs that connect these contigs. Gap filling of the scaffolds was performed using paired-end reads and the obtained sequences with the lowest numbers of Ns, until the process could not be extended on either end; the resulting sequences were called unigenes.

To gain protein function annotation information for the unigenes, BLASTX alignment (e-value < 10^−5^) was first conducted between unigenes and protein databases, such as Nr, Swiss-Prot, KEGG, and COG, and the unigene sequences were then searched against the Nt database using BLASTN. According to the Nr annotation information, the GO annotation information for the unigenes was obtained using the Blast2GO program. GO functional classification of the unigenes was performed using the WEGO software after obtaining GO annotation information for all of the unigenes.

### Detection of the EST-SSR markers and primer design

SSRs were detected in the assembled unigenes using the Simple Sequence Repeat Identification Tool program (MicroSatellite, http://www.gramene.org/db/markers/ssrtool)^51^; the SSRs were considered to contain mono-, di-, tri-, tetra-, penta-, and hexa-nucleotides with minimum repeat numbers of 12, six, five, five, four, and four, respectively. The EST-SSR primers were designed using BatchPrimer3 (http://probes.pw.usda.gov/cgi-bin/batchprimer3/batchprimer3.cgi)^13^,^51^, and the designed EST-SSR primers were synthesised by Shanghai Sangon Biological Engineering Technology (Shanghai, China).

### EST-SSR amplification and diversity analysis

A total of 15 accessions of *E. sibiricus* (Table S3), which were obtained the southern Gansu Province and northwestern plateau of Sichuan Province, were selected for polymorphism analyses with the EST-SSRs, and each accession contains three individual plants. Genomic DNA was separately isolated from the young leaves of three individual plants in each accession using the modified cetyltrimethylammonium bromide (CTAB) method^60^. The quantity and quality of the DNA samples used for PCR amplification were determined using a NanoDrop ND1000 spectrophotometer (Thermo Scientific, USA), and the concentration of each sample was adjusted to 50 ng/μl. PCR amplifications were performed in a final volume of 10 μL containing 40 ng of template DNA, 1 × PCR buffer, 2.0 mM MgCl_2_, 2.5 mM dNTPs, primers (4 μM each), and 0.8 U of Taq polymerase (TaKaRa, Kyoto, Japan)^14^. The PCR amplification conditions were as follows: initial denaturation at 94 °C for three min followed by 35 cycles of 30 s at 94 °C, 30 s at the annealing temperature (*Tm*), and 20 s at 72 °C and a final extension of seven min at 72 °C. The PCR products were subjected to electrophoresis on 8.0% non-denaturing polyacrylamide gels and stained using nucleic acid dye (Lot# I20826, GelStain, China). In addition, the DL500 DNA marker (TaKaRa, Kyoto, Japan) was used to determine the sizes of the PCR products. The number of alleles and the *Ho*, *He*, and *PIC* values were calculated as previously described^61^. Cluster analysis was performed to generate a dendrogram using UPGMA and Nei’s unbiased genetic distance with the FreeTree program and the TreeView software package^29^. Bootstrap values were obtained by 1000 replicate resamplings of replacements over the loci.

### Cross-species amplification

Thirteen species of the genus *Elymus* were chosen to evaluate the transferability of these newly developed EST-SSR markers to other related species (Table S6), and each species contains three individual plants. These species were provided by the U.S. National Plant Germplasm System (NPGS). In addition, Pop3 and Pop12, which appear in Table S3, were selected as controls. The extraction of genomic DNA, PCR amplification and diversity analysis were performed as described above.

## Additional Information

**How to cite this article**: Zhou, Q. *et al.* Development and cross-species transferability of EST-SSR markers in Siberian wildrye (Elymus sibiricus L.) using Illumina sequencing. *Sci. Rep.* **6**, 20549; doi: 10.1038/srep20549 (2016).

## Supplementary Material

Supplementary Information

## Figures and Tables

**Table 1:** Summary of the analysis of *de novo* assembled EST-SSRs for *Elymus sibiricus* L.

Category	Items	Number
Raw Reads	Total Raw Reads	84,905,976
Clean Reads	Total Clean Reads	76,686,804
	Total Clean Nucleotides (nt)	6,901,812,360
	Q20 Percentage	97.97%
	N Percentage	0.00%
	GC Percentage	54.70%
Contigs	Total Number	246,164
	Total Length (bp)	65,849,844
	Mean Length (bp)	268
	N50 (bp)	356
Unigenes	Total Number	94,458
	Total Length (bp)	60,972,579
	Mean Length (bp)	645
	N50 (bp)	942
	Distinct Clusters	42,058
	Distinct Singletons	52,400
EST-SSRs	Total Number of Examined Sequences	94,458
	Total Number of Identified SSRs	8,769
	Number of SSR-Containing Sequences	7,732
	Number of Sequences Containing More Than One SSR	902

**Table 2:** Functional annotation of the *E. sibiricus* transcriptome.

Category	Number	Percentage (%)
Nr annotation	62,046	65.69
Nt annotation	69,881	73.98
Swiss-Prot annotation	38,846	41.13
KEGG annotation	36,118	38.24
COG annotation	21,406	22.66
GO annotation	41,711	44.16
All	75,384	79.81

**Table 3:** Length distribution of the EST-SSRs based on the number of nucleotide repeat units.

Number of repeats	Mono-	Di-	Tri-	Quad-	Penta-	Hexa-	Total	Percentage (%)
4	0	0	0	0	381	191	572	6.52
5	0	0	3,502	261	45	0	3808	43.43
6	0	898	1,300	42	0	0	2240	25.54
7	0	432	478	0	0	0	910	10.38
8	0	269	38	0	0	0	307	3.50
9	0	183	1	0	0	0	184	2.10
10	0	139	0	0	0	0	139	1.59
11	0	149	0	0	0	0	149	1.70
12	118	16	0	0	0	0	134	1.53
13	88	0	0	0	0	0	88	1.00
14	64	0	0	0	0	0	64	0.73
15	52	0	0	0	0	0	52	0.59
16	21	0	0	0	0	0	21	0.24
17	15	0	0	0	0	0	15	0.17
18	11	0	0	0	0	0	11	0.13
19	7	0	0	0	0	0	7	0.08
20	12	0	0	0	0	0	12	0.14
21	11	0	0	0	0	0	11	0.13
22	23	0	0	0	0	0	23	0.26
23	20	0	0	0	0	0	20	0.23
24	2	0	0	0	0	0	2	0.02
Total	444	2086	5319	303	426	191	8769	
Percentage (%)	5.06	23.79	60.66	3.46	4.86	2.18		

## References

[b1] Dewey D. R. In Gene Manipulation In Plant Improvement (ed. Gustafson J. P.). 209–279 (Plenum Publishing Corp, 1984).

[b2] Dou Q. W., Zhang T. L. & Tsujimoto H. Physical mapping of repetitive sequences and genome analysis in six *Elymus* species by *in situ* hybridization. J. Syst. Evol. 49, 347–352 (2011).

[b3] Ma X., Chen S. Y., Zhang X. Q., Bai S. Q. & Zhang C. B. Assessment of worldwide genetic diversity of Siberian wildrye (*Elymus sibiricus* L.) germplasm based on gliadin analysis. Molecules 17, 4424–4434 (2012). 22499189 10.3390/molecules17044424 PMC6268020

[b4] Lei Y. T., Zhao Y. Y., Yu F., Li Y. & Dou Q. W. Development and characterization of 53 polymorphic genomic-SSR markers in Siberian wildrye (Elymus sibiricus L.). Conserv. Genet. Resour. 6, 861–864 (2014).

[b5] Sun G. L., Salomon B. & Bothmer R. V. Microsatellite polymorphism and genetic differentiation in three Norwegian populations of *Elymus alaskanus* (Poaceae). Plant. Syst. Evol. 234, 101–110 (2002).

[b6] Song Y. P. *et al.* Differences of EST-SSR and genomic-SSR markers in assessing genetic diversity in poplar. Forestry Studies in China 14, 1–7 (2012).

[b7] Naghavi M. R. *et al.* Comparison of genetic variation among accessions of *Aegilops tauschii* using AFLP and SSR markers. Genet. Resour. Crop Ev. 54, 237–240 (2007).

[b8] Gupta P. K., Langridge P. & Mir R. R. Marker-assisted wheat breeding: present status and future possibilities. Mol. Breeding 26, 145–161 (2010).

[b9] Prasanna B. M., Pixley K., Warburton M. L. & Xie C. X. Molecular marker-assisted breeding options for maize improvement in Asia. Mol. Breeding 26, 339–356 (2010).

[b10] Salem K. F. M., Varshney R. K., Röder M. S. & Börner A. EST-SSR based estimates on functional genetic variation in a barley (*Hordeum vulgare* L.) collection from Egypt. Genet. Resour. Crop Ev. 57, 515–521 (2010).

[b11] Li H. T. *et al.* Development and genetic mapping of microsatellite markers from whole genome shotgun sequences in *Brassica oleracea*. Mol. Breeding 28, 585–596 (2011).

[b12] Wu J., Cai C. F., Cheng F. Y., Cui H. L. & Zhou H. Characterisation and development of EST-SSR markers in tree peony using transcriptome sequences. Mol. Breeding 34, 1853–1866 (2014).

[b13] Liu Z. P. *et al.* Global transcriptome sequencing using the Illumina platform and the development of EST-SSR markers in autotetraploid alfalfa. PloS One 8, e83549 (2013). 24349529 10.1371/journal.pone.0083549 PMC3861513

[b14] Zhou Q., Chen T. L., Wang Y. R. & Liu Z. P. The development of 204 novel EST-SSRs and their use for genetic diversity analyses in cultivated alfalfa. Biochem. Syst. Ecol. 57, 227–230 (2014).

[b15] Liu Z. P., Liu P., Luo D., Liu W. X. & Wang Y. R. Exploiting Illumina sequencing for the development of 95 novel polymorphic EST-SSR markers in common vetch (*Vicia sativa* subsp. *sativa*). Molecules 19, 5777–5789 (2014). 24802988 10.3390/molecules19055777 PMC6271487

[b16] Surapaneni M., Yepuri V., Vemireddy L. R., Ghanta A. & Siddiq E. A. Development and characterization of microsatellite markers in Indian sesame (*Sesamum indicum* L.). Mol. Breeding 34, 1185–1200 (2014).

[b17] Sun G. L., Salomon B. & Bothmer R. V. Characterization and analysis of microsatellite loci in *Elymus caninus* (Triticeae: Poaceae). Theor. Appl. Genet. 96, 676–682 (1998).

[b18] MacRitchie D. & Sun G. Evaluating the potential of barley and wheat microsatellite markers for genetic analysis of *Elymus trachycaulus* complex species. Theor. Appl. Genet. 108, 720–724 (2004). 14556051 10.1007/s00122-003-1472-0

[b19] Xie W. G., Zhao X. H., Zhang J. Q., Wang Y. R. & Liu W. X. Assessment of genetic diversity of Siberian wild rye (*Elymus sibiricus* L.) germplasms with variation of seed shattering and implication for future genetic improvement. Biochem. Syst. Ecol. 58, 211–218 (2015).

[b20] Koilkonda P. *et al.* Large-scale development of expressed sequence tag-derived simple sequence repeat markers and diversity analysis in *Arachis* spp. Mol. Breeding 30, 125–138 (2012). 10.1007/s11032-011-9604-8 PMC3362703 22707912

[b21] Jia X. P., Deng Y. M., Sun X. B., Liang L. J. & Ye X. Q. Characterization of the global transcriptome using Illumina sequencing and novel microsatellite marker information in seashore paspalum. Genes Genom. 37, 77–86 (2015).

[b22] Chen P. *et al.* Transcriptome *de novo* assembly and differentially expressed genes related to cytoplasmic male sterility in kenaf (*Hibiscus cannabinus* L.). Mol. Breeding 34, 1879–1891 (2014).

[b23] Wei F. *et al.* Transcriptome sequencing and comparative analysis reveal long-term flowing mechanisms in *Hevea brasiliensis* latex. Gene 556, 153–162 (2015). 25431836 10.1016/j.gene.2014.11.048

[b24] Zhang G. J. *et al.* Deep RNA sequencing at single base-pair resolution reveals high complexity of the rice transcriptome. Genome Res. 20, 646–654 (2010). 20305017 10.1101/gr.100677.109 PMC2860166

[b25] Postnikova O. A., Shao J. & Nemchinov L. G. Analysis of the alfalfa root transcriptome in response to salinity stress. Plant Cell Physiol. 54, 1041–1055 (2013). 23592587 10.1093/pcp/pct056

[b26] Liu Z. P., Ma L. C., Nan Z. B. & Wang Y. R. Comparative transcriptional profiling provides insights into the evolution and development of the zygomorphic flower of *Vicia sativa* (Papilionoideae). PloS One 8, e57338 (2013). 23437373 10.1371/journal.pone.0057338 PMC3578871

[b27] Tombuloglu G., Tombuloglu H., Sakcali M. S. & Unver T. High-throughput transcriptome analysis of barley (*Hordeum vulgare*) exposed to excessive boron. Gene 557, 71–81 (2015). 25498907 10.1016/j.gene.2014.12.012

[b28] Du Z., Zhou X., Ling Y., Zhang Z. H. & Su Z. AgriGO: a GO analysis toolkit for the agricultural community. Nucleic Acids Res. 38, 64–70 (2010). 10.1093/nar/gkq310 PMC2896167 20435677

[b29] Pavlícek A., Hrdá S. & Flegr J. Free-Tree-Freeware program for construction of phylogenetic trees on the basis of distance data and bootstrap/jackknife analysis of the tree robustness. Application in the RAPD analysis of genus *Frenkelia*. Folia Biol. 45, 97–99 (1999). 10730897

[b30] Wang Z., Gerstein M. & Snyder M. RNA-Seq: a revolutionary tool for transcriptomics. Nat. Rev. Genet. 10, 57–63 (2009). 19015660 10.1038/nrg2484 PMC2949280

[b31] Guo R. *et al.* Characterization and cross-species transferability of EST-SSR markers developed from the transcriptome of *Dysosma versipellis* (Berberidaceae) and their application to population genetic studies. Mol. Breeding 34, 1733–1746 (2014).

[b32] Tan L. Q. *et al.* Floral transcriptome sequencing for SSR marker development and linkage map construction in the tea plant (*Camellia sinensis*). PloS One 8, e81611 (2013). 24303059 10.1371/journal.pone.0081611 PMC3841144

[b33] Wang Z. Y. *et al.* *De novo* assembly and characterization of root transcriptome using Illumina paired-end sequencing and development of cSSR markers in sweet potato (*Ipomoea batatas*). BMC genomics 11, 726 (2010). 21182800 10.1186/1471-2164-11-726 PMC3016421

[b34] Zhang J. X. *et al.* Transcriptome analysis of *Cymbidium sinense* and its application to the identification of genes associated with floral development. BMC genomics 14, 279 (2013). 23617896 10.1186/1471-2164-14-279 PMC3639151

[b35] Wei W. L. *et al.* Characterization of the sesame (*Sesamum indicum* L.) global transcriptome using Illumina paired-end sequencing and development of EST-SSR markers. BMC genomics 12, 451 (2011). 21929789 10.1186/1471-2164-12-451 PMC3184296

[b36] Jiang B., Xie D. S., Liu W. R., Peng Q. W. & He X. M. *De novo* assembly and characterization of the transcriptome, and development of SSR markers in wax gourd (*Benicasa hispida*). PloS One 8, e71054 (2013). 23951078 10.1371/journal.pone.0071054 PMC3738631

[b37] Li C. *et al.* *De novo* assembly and characterization of fruit transcriptome in *Litchi chinensis* Sonn and analysis of differentially regulated genes in fruit in response to shading. BMC genomics 14, 552 (2013). 23941440 10.1186/1471-2164-14-552 PMC3751308

[b38] Ong W. D., Voo C. L. Y. & Kumar S. V. Development of ESTs and data mining of pineapple EST-SSRs. Mol. Biol. Rep. 39, 5889–5896 (2012). 22207174 10.1007/s11033-011-1400-3

[b39] Pan L. *et al.* Development of novel EST-SSRs from sacred lotus (*Nelumbo nucifera* Gaertn) and their utilization for the genetic diversity analysis of *N. nucifera*. J. Hered. 101, 71–82 (2009). 19666746 10.1093/jhered/esp070

[b40] Jena S. N. *et al.* Development and characterization of genomic and expressed SSRs for levant cotton (*Gossypium herbaceum* L.). Theor. Appl. Genet. 124, 565–576 (2012). 22038488 10.1007/s00122-011-1729-y

[b41] Qiu L. J., Yang C., Tian B., Yang J. B. & Liu A. Z. Exploiting EST databases for the development and characterization of EST-SSR markers in castor bean (*Ricinus communis* L.). BMC Plant Biol. 10, 278 (2010). 21162723 10.1186/1471-2229-10-278 PMC3017068

[b42] Wang S. F. *et al.* Transcriptome analysis of the roots at early and late seedling stages using Illumina paired-end sequencing and development of EST-SSR markers in radish. Plant Cell Rep. 31, 1437–1447 (2012). 22476438 10.1007/s00299-012-1259-3

[b43] Gong L. & Deng Z. EST-SSR markers for gerbera (*Gerbera hybrida*). Mol. Breeding 26, 125–132 (2010).

[b44] Varshney R. K., Graner A. & Sorrells M. E. Genic microsatellite markers in plants: features and applications. Trends Biotechnol. 23, 48–55 (2005). 15629858 10.1016/j.tibtech.2004.11.005

[b45] Biswas M. K. *et al.* Exploiting BAC-end sequences for the mining, characterization and utility of new short sequences repeat (SSR) markers in Citrus. Mol. Biol. Rep. 39, 5373–5386 (2012). 22170603 10.1007/s11033-011-1338-5

[b46] Li D. J., Deng Z., Qin B., Liu X. H. & Men Z. H. *De novo* assembly and characterization of bark transcriptome using Illumina sequencing and development of EST-SSR markers in rubber tree (*Hevea brasiliensis* Muell. Arg.). BMC genomics 13, 192 (2012). 22607098 10.1186/1471-2164-13-192 PMC3431226

[b47] Zhai L. L. *et al.* Novel and useful genic-SSR markers from *de novo* transcriptome sequencing of radish (*Raphanus sativus* L.). Mol. Breeding 33, 611–624 (2014).

[b48] Luo D. *et al.* Novel polymorphic expressed-sequence tag-simple-sequence repeat markers in *Campeiostachys nutans* for genetic diversity analyses. Crop Sci. 55, 2712–2718 (2015).

[b49] Wang Z., Yan H. W., Fu X. N., Li X. H. & Gao H. W. Development of simple sequence repeat markers and diversity analysis in alfalfa (*Medicago sativa* L.). Mol. Biol. Rep. 40, 3291–3298 (2013). 23275197 10.1007/s11033-012-2404-3

[b50] Feng S. P., Li W. G., Huang H. S., Wang J. Y. & Wu Y. T. Development, characterization and cross-species/genera transferability of EST-SSR markers for rubber tree (*Hevea brasiliensis*). Mol. Breeding 23, 85–97 (2009).

[b51] Zheng X. F. *et al.* Development of microsatellite markers by transcriptome sequencing in two species of *Amorphophallus* (Araceae). BMC genomics 14, 490 (2013). 23870214 10.1186/1471-2164-14-490 PMC3737116

[b52] Thiel T., Michalek W., Varshney R. & Graner A. Exploiting EST databases for the development and characterization of gene-derived SSR-markers in barley (*Hordeum vulgare* L.). Theor. Appl. Genet. 106, 411–422 (2003). 12589540 10.1007/s00122-002-1031-0

[b53] Ma X., Zhang X. Q., Zhou Y. H., Bai S. Q. & Liu W. Assessing genetic diversity of *Elymus sibiricus* (Poaceae: Triticeae) populations from Qinghai-Tibet plateau by ISSR markers. Biochem. Syst. Ecol. 36, 514–522 (2008).

[b54] Zhang J. C., Xie W. G., Wang Y. R. & Zhao X. H. Potential of start codon targeted (SCoT) markers to estimate genetic diversity and relationships among chinese *Elymus sibiricus* accessions. Molecules 20, 5987–6001 (2015). 25853316 10.3390/molecules20045987 PMC6272172

[b55] Xu P. *et al.* Partial sequencing of the bottle gourd genome reveals markers useful for phylogenetic analysis and breeding. BMC genomics 12, 467 (2011). 21942996 10.1186/1471-2164-12-467 PMC3188536

[b56] Fernandez-Silva I. *et al.* Bin mapping of genomic and EST-derived SSRs in melon (*Cucumis melo* L.). Theor. Appl. Genet. 118, 139–150 (2008). 18806992 10.1007/s00122-008-0883-3

[b57] Liu Q. L. *et al.* Phylogenetic relationships in *Elymus* (Poaceae: Triticeae) based on the nuclear ribosomal internal transcribed spacer and chloroplast *trnL-F* sequences. New Phytol. 170, 411–420 (2006). 16608465 10.1111/j.1469-8137.2006.01665.x

[b58] Mason-Gamer R. J. Allopolyploids of the genus *Elymus* (Triticeae, Poaceae): a phylogenetic perspective. Aliso 23, 372–379 (2007).

[b59] Mason-Gamer R. J. Phylogeny of a genomically diverse group of *Elymus* (Poaceae) allopolyploids reveals multiple levels of reticulation. PloS One 8, e78449 (2013). 24302986 10.1371/journal.pone.0078449 PMC3840256

[b60] Doyle J. J. & Doyle J. L. A rapid DNA isolation procedure for small quantities of fresh leaf tissue. Phytochem Bull 19, 11–15 (1987).

[b61] Chung J. W., Kim T. S., Suresh S., Lee S. Y. & Cho G. T. Development of 65 novel polymorphic cDNA-SSR markers in common vetch (*Vicia sativa* subsp*. sativa*) using next generation sequencing. Molecules 18, 8376–8392 (2013). 23863776 10.3390/molecules18078376 PMC6270072

