# Supplementary material for: Development and cross-species transferability of EST-SSR markers in Siberian wildrye (Elymus sibiricus L.) using Illumina sequencing
Source: Sci Rep. 2016 Feb 8;6:20549. doi: 10.1038/srep20549 (PMC4744933; doi:10.1038/srep20549)
Supplement: Supplementary Information [file srep20549-s1.pdf]

Development and cross-species transferability of EST-SSR markers in Siberian wildrye (*Elymus sibiricus* L.) using Illumina sequencing

Qiang Zhou, Dong Luo, Lichao Ma, Wengang Xie, Yu Wang, Yanrong Wang and Zhipeng Liu*

The State Key Laboratory of Grassland Agro-ecosystems, College of Pastoral Agriculture Science and Technology, Lanzhou University, Lanzhou, 730020, China
E-mail: lzp@lzu.edu.cn

Supplemental Figure S1-S9
Supplemental Table S1-S6

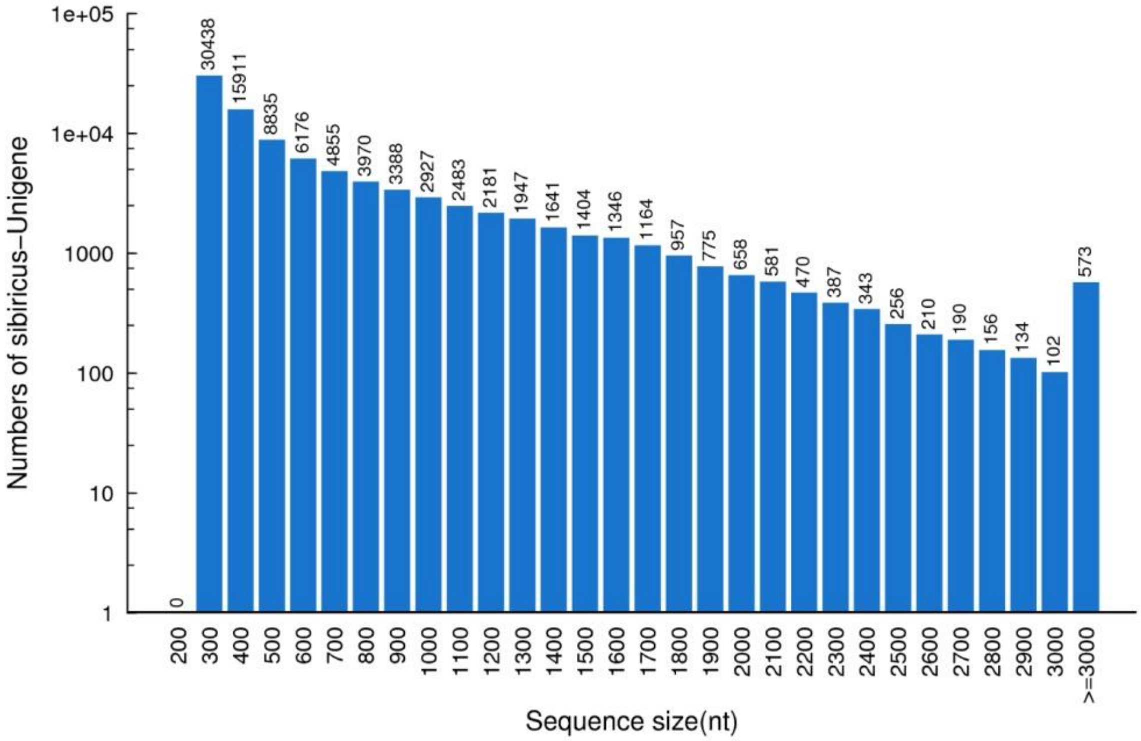

Supplementary Fig. S1: Length distribution of all unigenes. The x-axis represents the size of all unigenes, and the y-axis represents the number of all unigenes with a certain length.

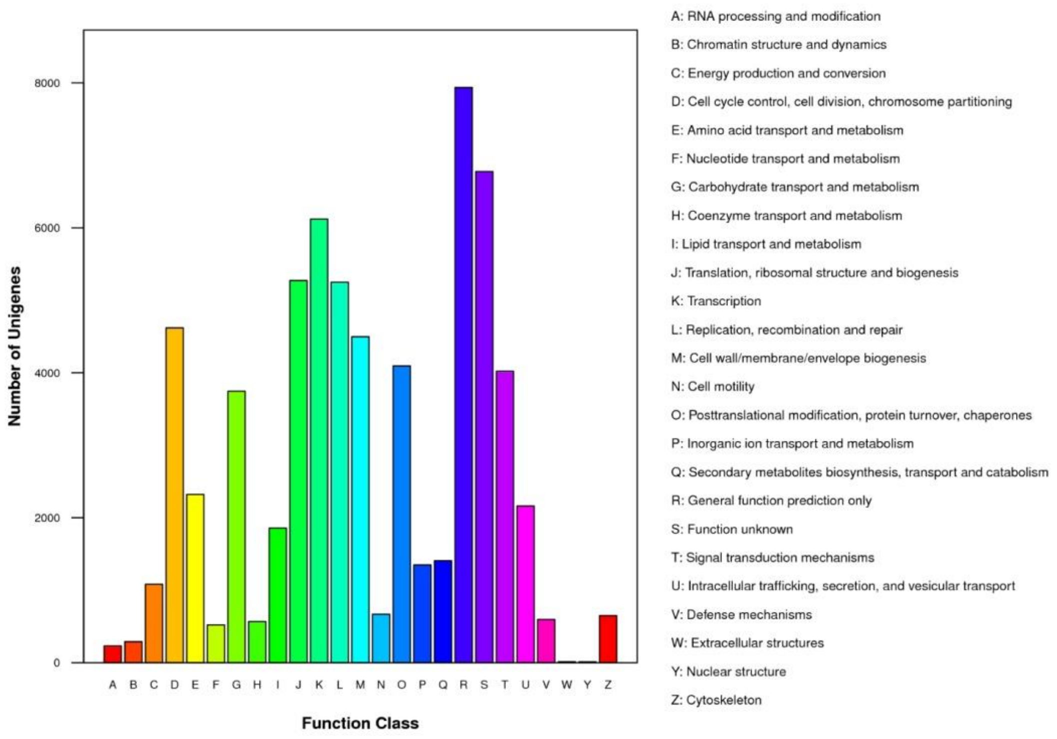

Supplementary Fig. S2: COG analysis of the unigene sequences. The y-axis indicates the number of unigenes in a specific functional cluster.

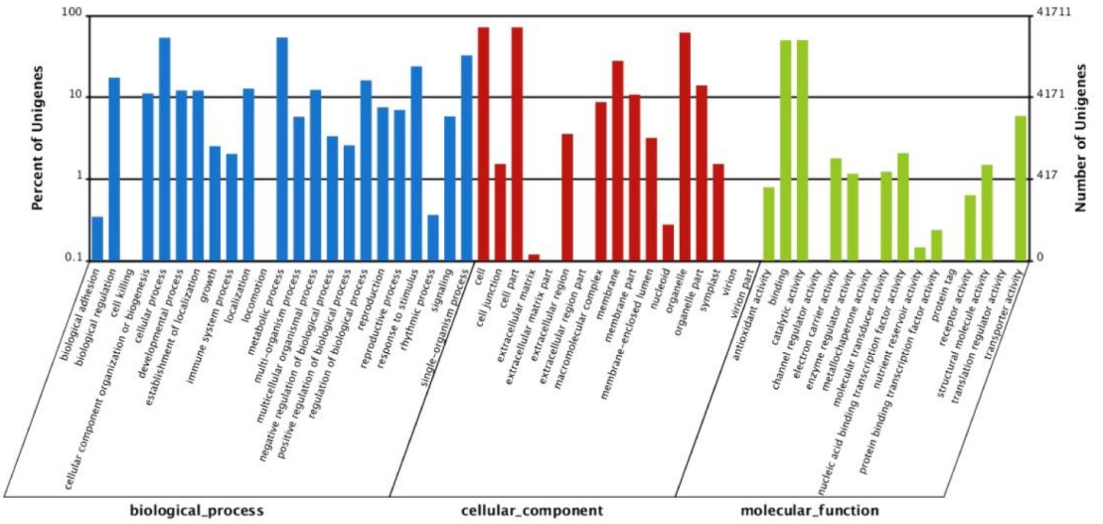

Supplementary Fig. S3: Summary of GO analysis of the unigene sequences. The y-axis on the right indicates the number of genes in a category. The y-axis on the left indicates the percentage of a specific category of genes in that main category.

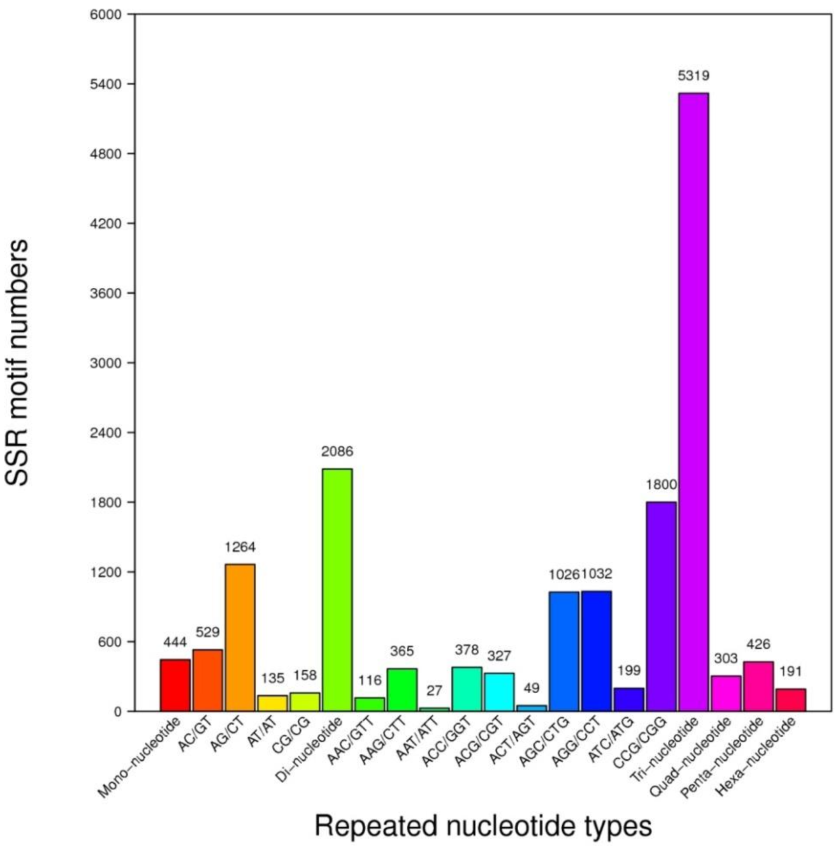

Supplementary Fig. S4: Statistics of the SSR length distribution. In total, 317 types of motifs were identified in the EST-SSRs.

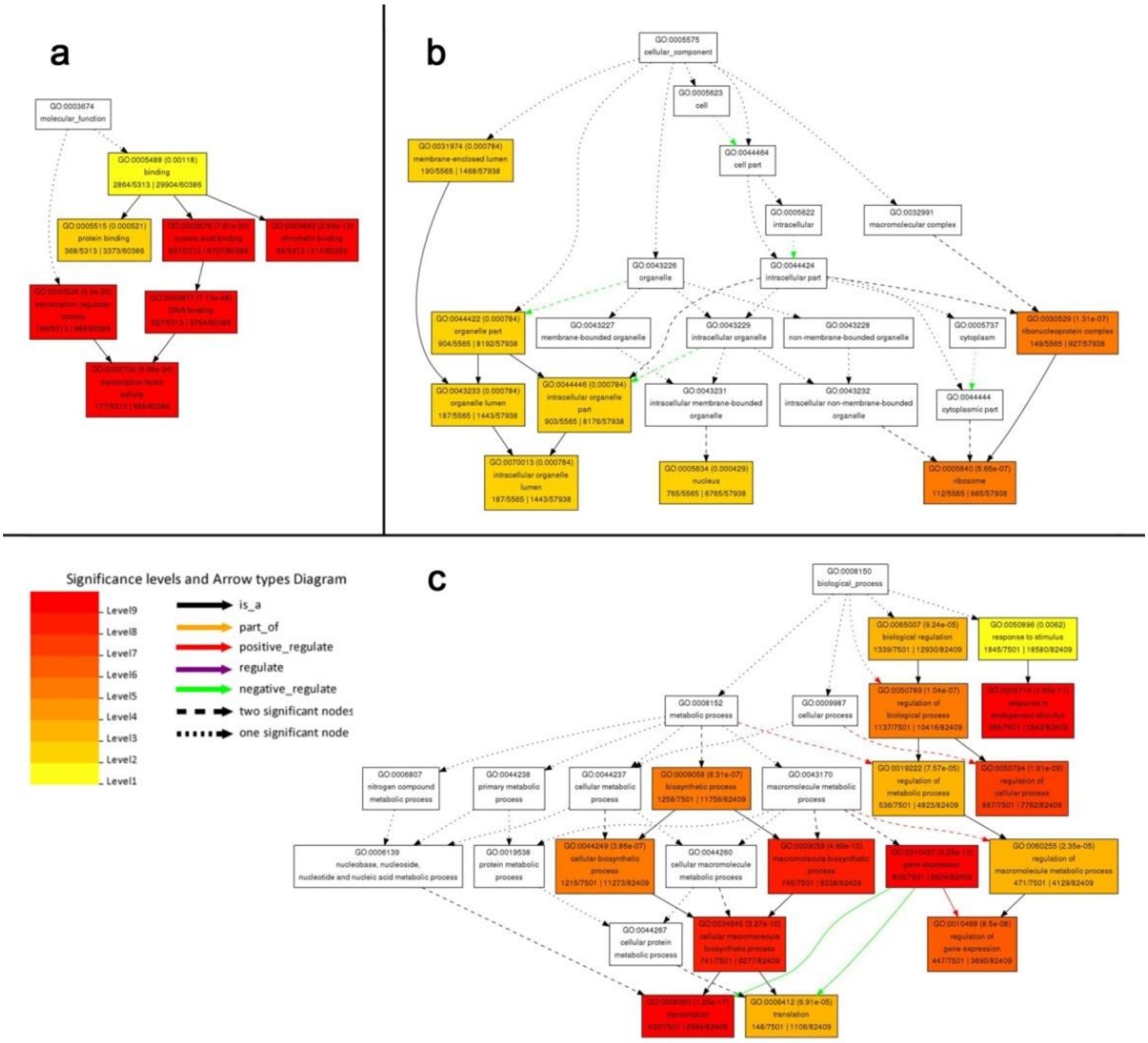

Supplementary Fig. S5: Gene ontology (GO) term enrichment status for SSR repeat-containing unigenes in *E. sibiricus*. (a) Molecular function. (b) Cellular component. (c) Biological process. The scale shows the p-value cut-off levels for each biological process. A deeper colour denotes a more significant biological pathway.

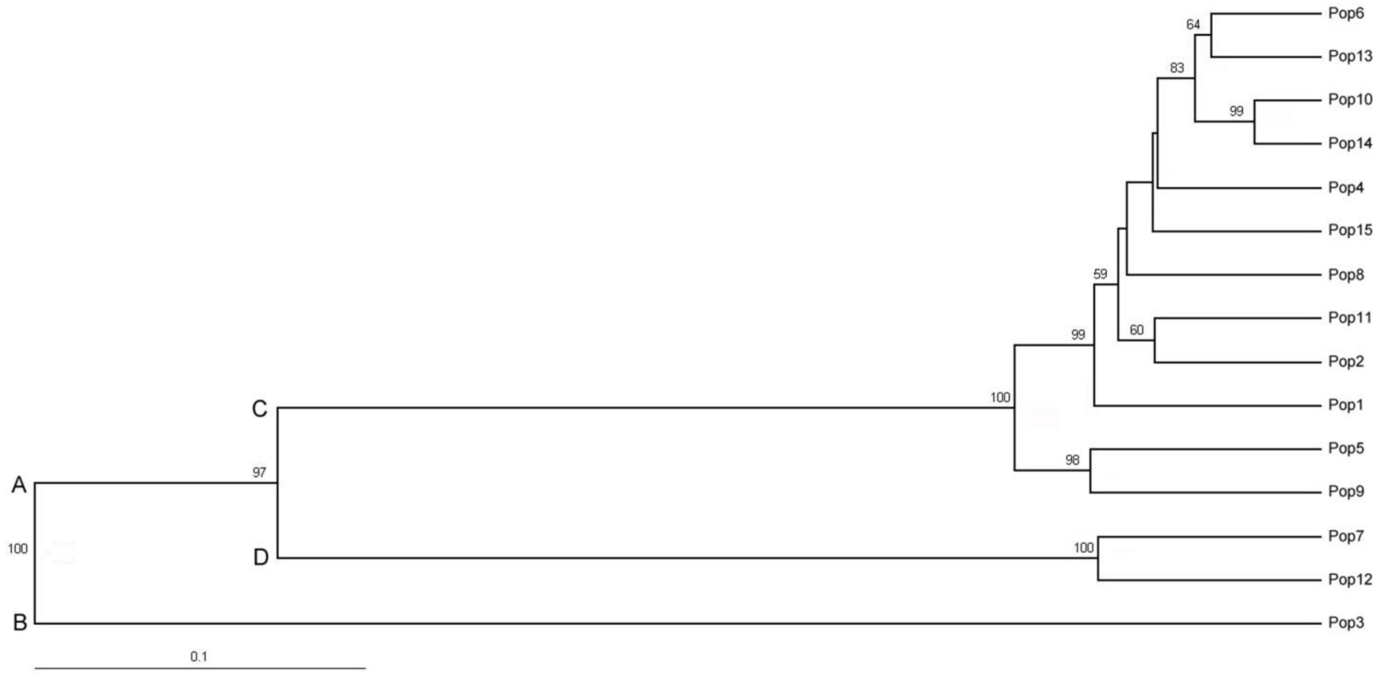

Supplementary Fig. S6: Dendrogram of 15 *E. sibiricus* accessions based on 112 EST-SSR primer pairs. Pop 1 through Pop 15 represent the 15 *E. sibiricus* accessions. The corresponding detailed information for the 15 *E. sibiricus* accessions is shown in Supplementary Table S3. And the bootstrap values over 50% are showed.

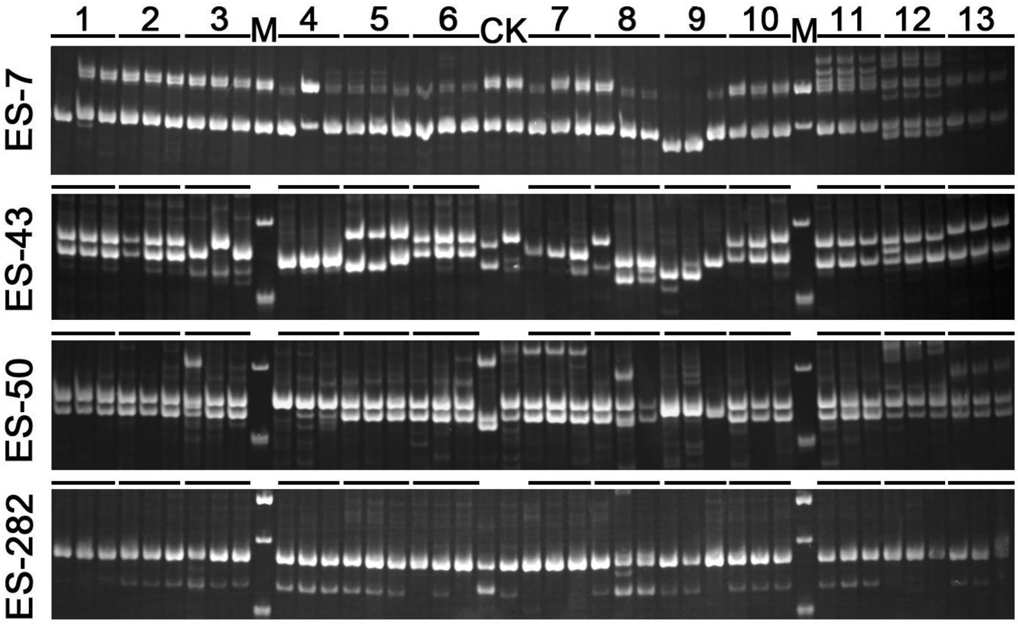

Supplementary Fig. S7: EST-SSR marker variations at the ES-7, ES-43, ES-50, and ES-282 loci of 13 species of the *Elymus* genus. Each species includes three individual plants; 'CK' represents *E. sibiricus* and the letter 'M' denotes the molecular markers, which are 200 bp and 150 bp (top to bottom) in ES-7, 150 bp and 100 bp in ES-43 and ES-50 (top to bottom), and 200 bp, 150 bp, and 100 bp (top to bottom) in ES-282.

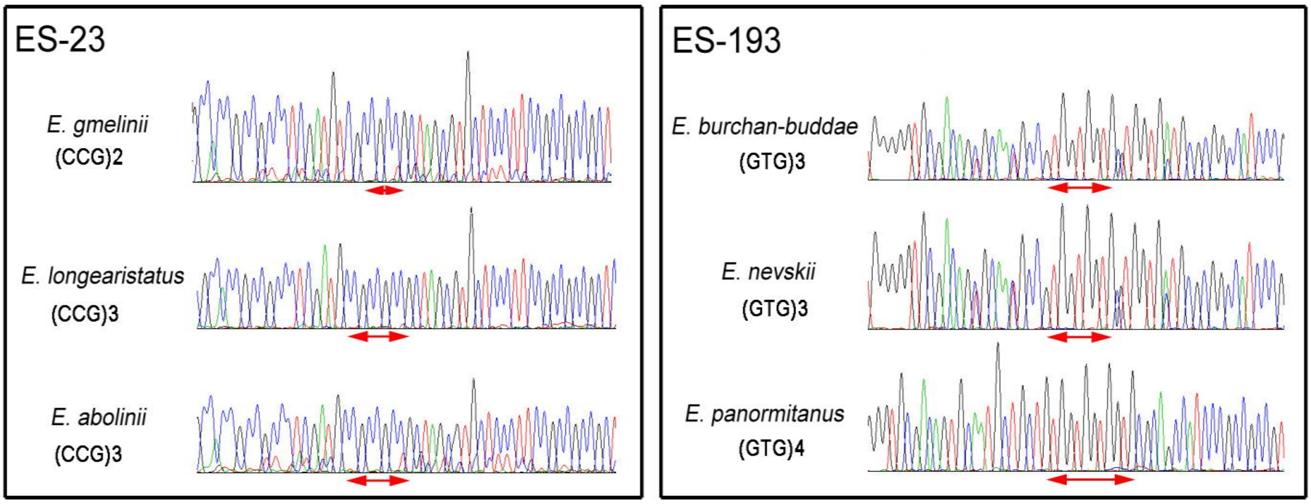

Supplementary Fig. S8: Comparative electropherogram analysis of two EST-SSR loci (ES-23 and ES-193) among different species of *Elymus*.

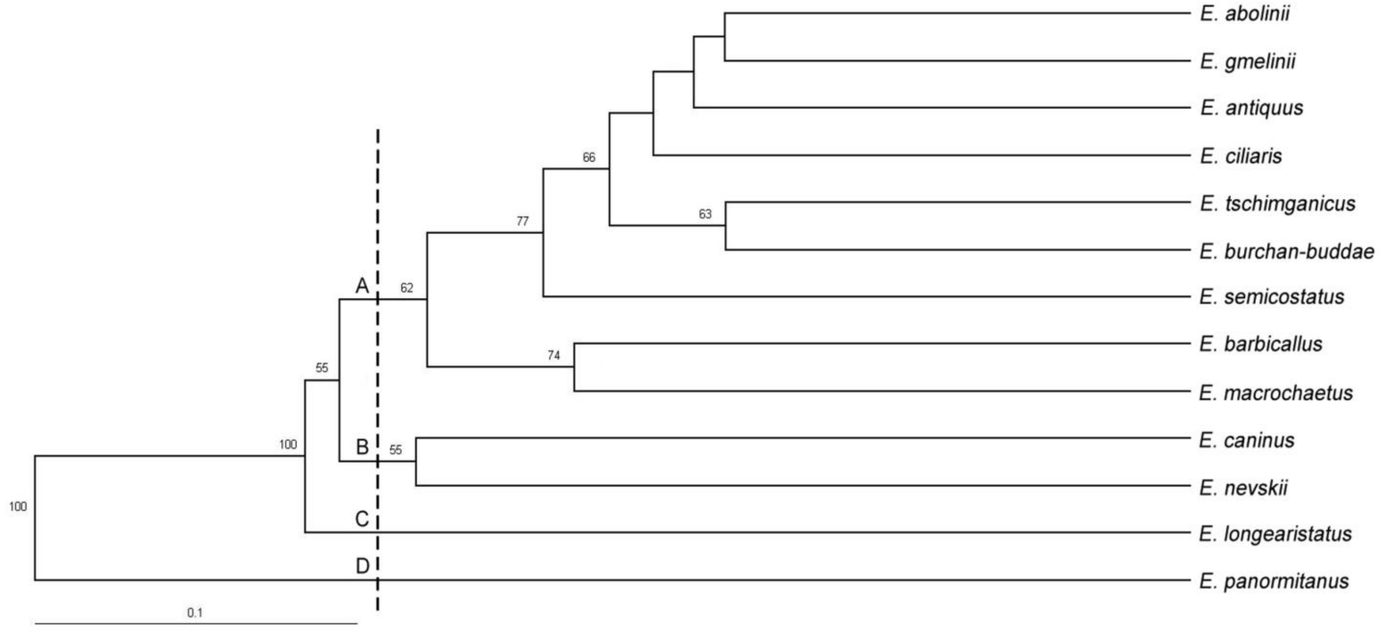

Supplementary Fig. S9: Cluster analysis of 13 species of the *Elymus* genus based on 55 EST-SSR primer pairs. The corresponding detailed information for the 13 species of the genus *Elymus* is shown in Supplementary Table S6. And the bootstrap values over 50% are showed.

Supplementary Table S1: Detailed information of EST-SSRs based on the number of nucleotide repeat units.

Repeats	4	5	6	7	8	9	10	11	12	13	14	≥15	Total	Percentage (%)
A/T	-	-	-	-	-	-	-	-	108	79	60	148	395	4.50
C/G	-	-	-	-	-	-	-	-	10	9	4	26	49	0.56
AC/GT	-	-	216	93	65	63	36	47	9	-	-	-	529	6.03
AG/CT	-	-	488	272	189	110	103	97	5	-	-	-	1264	14.41
AT/AT	-	-	71	38	11	8	-	5	2	-	-	-	135	1.54
CG/CG	-	-	123	29	4	2	-	-	-	-	-	-	158	1.80
AAC/GTT	-	71	20	24	1	-	-	-	-	-	-	-	116	1.32
AAG/CTT	-	218	77	69	1	-	-	-	-	-	-	-	365	4.16
AAT/ATT	-	21	4	2	-	-	-	-	-	-	-	-	27	0.31
ACC/GGT	-	252	86	32	8	-	-	-	-	-	-	-	378	4.31
ACG/CGT	-	211	72	42	2	-	-	-	-	-	-	-	327	3.73
ACT/AGT	-	35	8	4	2	-	-	-	-	-	-	-	49	0.56
AGC/CTG	-	659	269	96	2	-	-	-	-	-	-	-	1026	11.70
AGG/CCT	-	704	241	73	14	-	-	-	-	-	-	-	1032	11.77
ATC/ATG	-	123	54	17	4	1	-	-	-	-	-	-	199	2.27
CCG/CGG	-	1208	469	119	4	-	-	-	-	-	-	-	1800	20.53
AAAC/GTTT	-	4	1	-	-	-	-	-	-	-	-	-	5	0.06
AAAG/CTTT	-	12	2	-	-	-	-	-	-	-	-	-	14	0.16
AAAT/ATTT	-	1	2	-	-	-	-	-	-	-	-	-	3	0.03
AACC/GGTT	-	14	2	-	-	-	-	-	-	-	-	-	16	0.18
AACG/CGTT	-	4	-	-	-	-	-	-	-	-	-	-	4	0.05
AACT/AGTT	-	-	2	-	-	-	-	-	-	-	-	-	2	0.02
AAGC/CTTG	-	9	-	-	-	-	-	-	-	-	-	-	9	0.10
AAGG/CCTT	-	19	-	-	-	-	-	-	-	-	-	-	19	0.22
AATC/ATTG	-	7	-	-	-	-	-	-	-	-	-	-	7	0.08
AATG/ATTC	-	8	2	-	-	-	-	-	-	-	-	-	10	0.11
ACAG/CTGT	-	10	1	-	-	-	-	-	-	-	-	-	11	0.13
ACAT/ATGT	-	15	1	-	-	-	-	-	-	-	-	-	16	0.18
ACCG/CGGT	-	5	-	-	-	-	-	-	-	-	-	-	5	0.06
ACCT/AGGT	-	2	1	-	-	-	-	-	-	-	-	-	3	0.03
ACGC/CGTG	-	10	-	-	-	-	-	-	-	-	-	-	10	0.11
ACGG/CCGT	-	17	6	-	-	-	-	-	-	-	-	-	23	0.26
ACGT/ACGT	-	3	3	-	-	-	-	-	-	-	-	-	6	0.07
ACTC/AGTG	-	3	1	-	-	-	-	-	-	-	-	-	4	0.05
ACTG/AGTC	-	3	-	-	-	-	-	-	-	-	-	-	3	0.03
AGAT/ATCT	-	3	2	-	-	-	-	-	-	-	-	-	5	0.06
AGCC/CTGG	-	14	1	-	-	-	-	-	-	-	-	-	15	0.17
AGCG/CGCT	-	16	4	-	-	-	-	-	-	-	-	-	20	0.23
AGCT/AGCT	-	4	-	-	-	-	-	-	-	-	-	-	4	0.05

AGGC/CCTG	-	24	-	-	-	-	-	-	-	-	-	-	24	0.27
AGGG/CCCT	-	16	6	-	-	-	-	-	-	-	-	-	22	0.25
ATCC/ATGG	-	28	5	-	-	-	-	-	-	-	-	-	33	0.38
ATGC/ATGC	-	6	-	-	-	-	-	-	-	-	-	-	6	0.07
CCCG/CGGG	-	2	-	-	-	-	-	-	-	-	-	-	2	0.02
CCGG/CCGG	-	2	-	-	-	-	-	-	-	-	-	-	2	0.02
AAAAC/GTTTT	7	-	-	-	-	-	-	-	-	-	-	-	7	0.08
AAAAG/CTTTT	11	-	-	-	-	-	-	-	-	-	-	-	11	0.13
AAAAT/ATTTT	3	-	-	-	-	-	-	-	-	-	-	-	3	0.03
AAACC/GGTTT	11	1	-	-	-	-	-	-	-	-	-	-	12	0.14
AAACT/AGTTT	3	-	-	-	-	-	-	-	-	-	-	-	3	0.03
AAAGG/CCTTT	2	3	-	-	-	-	-	-	-	-	-	-	5	0.06
AAATC/ATTTG	4	-	-	-	-	-	-	-	-	-	-	-	4	0.05
AACAG/CTGTT	1	-	-	-	-	-	-	-	-	-	-	-	1	0.01
AACAT/ATGTT	1	-	-	-	-	-	-	-	-	-	-	-	1	0.01
AACCC/GGGTT	3	1	-	-	-	-	-	-	-	-	-	-	4	0.05
AACCG/CGGTT	1	-	-	-	-	-	-	-	-	-	-	-	1	0.01
AACGC/CGTTG	1	-	-	-	-	-	-	-	-	-	-	-	1	0.01
AACGG/CCGTT	-	1	-	-	-	-	-	-	-	-	-	-	1	0.01
AACTC/AGTTG	-	2	-	-	-	-	-	-	-	-	-	-	2	0.02
AACTG/AGTTC	1	-	-	-	-	-	-	-	-	-	-	-	1	0.01
AAGAG/CTCTT	13	2	-	-	-	-	-	-	-	-	-	-	15	0.17
AAGAT/ATCTT	2	-	-	-	-	-	-	-	-	-	-	-	2	0.02
AAGCC/CTTGG	4	-	-	-	-	-	-	-	-	-	-	-	4	0.05
AAGCT/AGCTT	1	1	-	-	-	-	-	-	-	-	-	-	2	0.02
AAGGC/CCTTG	1	-	-	-	-	-	-	-	-	-	-	-	1	0.01
AAGGG/CCCTT	8	12	-	-	-	-	-	-	-	-	-	-	20	0.23
AATAC/ATTGT	2	-	-	-	-	-	-	-	-	-	-	-	2	0.02
AATAG/ATTCT	4	-	-	-	-	-	-	-	-	-	-	-	4	0.05
AATCC/ATTGG	11	-	-	-	-	-	-	-	-	-	-	-	11	0.13
AATCG/ATTCG	1	1	-	-	-	-	-	-	-	-	-	-	2	0.02
AATGC/ATTGC	4	7	-	-	-	-	-	-	-	-	-	-	11	0.13
AATGG/ATTCC	2	-	-	-	-	-	-	-	-	-	-	-	2	0.02
AATGT/ACATT	1	-	-	-	-	-	-	-	-	-	-	-	1	0.01
ACACC/GGTGT	12	1	-	-	-	-	-	-	-	-	-	-	13	0.15
ACACG/CGTGT	1	-	-	-	-	-	-	-	-	-	-	-	1	0.01
ACAGC/CTGTG	6	-	-	-	-	-	-	-	-	-	-	-	6	0.07
ACATC/ATGTG	1	-	-	-	-	-	-	-	-	-	-	-	1	0.01
ACATG/ATGTC	-	1	-	-	-	-	-	-	-	-	-	-	1	0.01
ACCAG/CTGGT	6	-	-	-	-	-	-	-	-	-	-	-	6	0.07
ACCAT/ATGGT	2	1	-	-	-	-	-	-	-	-	-	-	3	0.03
ACCCC/GGGGT	5	-	-	-	-	-	-	-	-	-	-	-	5	0.06
ACCCG/CGGGT	2	-	-	-	-	-	-	-	-	-	-	-	2	0.02
ACCCT/AGGGT	1	-	-	-	-	-	-	-	-	-	-	-	1	0.01

ACCGC/CGGTG	4	-	-	-	-	-	-	-	-	-	-	-	4	0.05
ACCTC/AGGTG	4	-	-	-	-	-	-	-	-	-	-	-	4	0.05
ACCTG/AGGTC	4	1	-	-	-	-	-	-	-	-	-	-	5	0.06
ACGAG/CGTCT	5	-	-	-	-	-	-	-	-	-	-	-	5	0.06
ACGCC/CGTGG	6	1	-	-	-	-	-	-	-	-	-	-	7	0.08
ACGCG/CGCGT	11	-	-	-	-	-	-	-	-	-	-	-	11	0.13
ACGGC/CCGTG	3	-	-	-	-	-	-	-	-	-	-	-	3	0.03
ACGGG/CCCGT	6	-	-	-	-	-	-	-	-	-	-	-	6	0.07
ACTAG/AGTCT	2	-	-	-	-	-	-	-	-	-	-	-	2	0.02
ACTCC/AGTGG	6	-	-	-	-	-	-	-	-	-	-	-	6	0.07
ACTCG/AGTCG	4	-	-	-	-	-	-	-	-	-	-	-	4	0.05
ACTCT/AGAGT	1	1	-	-	-	-	-	-	-	-	-	-	2	0.02
ACTGG/AGTCC	3	-	-	-	-	-	-	-	-	-	-	-	3	0.03
AGAGC/CTCTG	27	-	-	-	-	-	-	-	-	-	-	-	27	0.31
AGAGG/CCTCT	38	-	-	-	-	-	-	-	-	-	-	-	38	0.43
AGATC/ATCTG	4	-	-	-	-	-	-	-	-	-	-	-	4	0.05
AGATG/ATCTC	5	1	-	-	-	-	-	-	-	-	-	-	6	0.07
AGCCC/CTGGG	4	-	-	-	-	-	-	-	-	-	-	-	4	0.05
AGCCG/CGGCT	2	-	-	-	-	-	-	-	-	-	-	-	2	0.02
AGCCT/AGGCT	4	-	-	-	-	-	-	-	-	-	-	-	4	0.05
AGCGC/CGCTG	2	-	-	-	-	-	-	-	-	-	-	-	2	0.02
AGCGG/CCGCT	2	-	-	-	-	-	-	-	-	-	-	-	2	0.02
AGCTC/AGCTG	6	1	-	-	-	-	-	-	-	-	-	-	7	0.08
AGGCG/CCTCG	18	1	-	-	-	-	-	-	-	-	-	-	19	0.22
AGGGC/CCCTG	14	-	-	-	-	-	-	-	-	-	-	-	14	0.16
AGGGG/CCCCT	28	2	-	-	-	-	-	-	-	-	-	-	30	0.34
ATCCC/ATGGG	11	-	-	-	-	-	-	-	-	-	-	-	11	0.13
ATCCG/ATCGG	1	-	-	-	-	-	-	-	-	-	-	-	1	0.01
ATCGC/ATGCG	3	2	-	-	-	-	-	-	-	-	-	-	5	0.06
ATGCC/ATGGC	1	-	-	-	-	-	-	-	-	-	-	-	1	0.01
CCCCG/CGGGG	5	1	-	-	-	-	-	-	-	-	-	-	6	0.07
CCCGG/CCGGG	1	-	-	-	-	-	-	-	-	-	-	-	1	0.01
CCGCG/CGCGG	7	-	-	-	-	-	-	-	-	-	-	-	7	0.08
AAAAAG/CTTTTT	2	-	-	-	-	-	-	-	-	-	-	-	2	0.02
AAAACC/GGTTTT	1	-	-	-	-	-	-	-	-	-	-	-	1	0.01
AAAAGG/CCTTTT	1	-	-	-	-	-	-	-	-	-	-	-	1	0.01
AAACCC/GGGTTT	2	-	-	-	-	-	-	-	-	-	-	-	2	0.02
AAAGAG/CTCTTT	2	-	-	-	-	-	-	-	-	-	-	-	2	0.02
AAAGAT/ATCTTT	3	-	-	-	-	-	-	-	-	-	-	-	3	0.03
AAATCC/ATTTGG	1	-	-	-	-	-	-	-	-	-	-	-	1	0.01
AAATCG/ATTTCG	2	-	-	-	-	-	-	-	-	-	-	-	2	0.02
AAATGC/ATTTGC	1	-	-	-	-	-	-	-	-	-	-	-	1	0.01
AAATGG/ATTTCC	1	-	-	-	-	-	-	-	-	-	-	-	1	0.01
AACAGC/CTGTTG	1	-	-	-	-	-	-	-	-	-	-	-	1	0.01

AACCCT/AGGGTT	1	-	-	-	-	-	-	-	-	-	-	-	1	0.01
AACCTC/AGGTTG	2	-	-	-	-	-	-	-	-	-	-	-	2	0.02
AACGCC/CGTTGG	2	-	-	-	-	-	-	-	-	-	-	-	2	0.02
AACGGC/CCGTTG	3	-	-	-	-	-	-	-	-	-	-	-	3	0.03
AACTGC/AGTTGC	1	-	-	-	-	-	-	-	-	-	-	-	1	0.01
AAGAGC/CTCTTG	1	-	-	-	-	-	-	-	-	-	-	-	1	0.01
AAGATG/ATCTTC	1	-	-	-	-	-	-	-	-	-	-	-	1	0.01
AAGCAC/CTTGTG	2	-	-	-	-	-	-	-	-	-	-	-	2	0.02
AAGCAG/CTGCTT	1	-	-	-	-	-	-	-	-	-	-	-	1	0.01
AAGCCC/CTTGGG	5	-	-	-	-	-	-	-	-	-	-	-	5	0.06
AAGCCG/CGGCTT	1	-	-	-	-	-	-	-	-	-	-	-	1	0.01
AAGCGG/CCGCTT	1	-	-	-	-	-	-	-	-	-	-	-	1	0.01
AAGGCG/CCTTCG	1	-	-	-	-	-	-	-	-	-	-	-	1	0.01
AAGGGG/CCCCTT	1	-	-	-	-	-	-	-	-	-	-	-	1	0.01
AAGGTG/ACCTTC	6	-	-	-	-	-	-	-	-	-	-	-	6	0.07
AATAGC/ATTGCT	3	-	-	-	-	-	-	-	-	-	-	-	3	0.03
AATCAG/ATTCTG	2	-	-	-	-	-	-	-	-	-	-	-	2	0.02
AATCCC/ATTGGG	2	-	-	-	-	-	-	-	-	-	-	-	2	0.02
AATCGG/ATTCCG	1	-	-	-	-	-	-	-	-	-	-	-	1	0.01
AATCTC/AGATTG	2	-	-	-	-	-	-	-	-	-	-	-	2	0.02
AATGAG/ATTCTC	2	-	-	-	-	-	-	-	-	-	-	-	2	0.02
AATGGC/ATTGCC	1	-	-	-	-	-	-	-	-	-	-	-	1	0.01
AATGGG/ATTCCC	1	-	-	-	-	-	-	-	-	-	-	-	1	0.01
ACACAG/CTGTGT	2	-	-	-	-	-	-	-	-	-	-	-	2	0.02
ACACCC/GGGTGT	1	-	-	-	-	-	-	-	-	-	-	-	1	0.01
ACACGC/CGTGTG	4	-	-	-	-	-	-	-	-	-	-	-	4	0.05
ACAGAG/CTCTGT	3	-	-	-	-	-	-	-	-	-	-	-	3	0.03
ACAGGC/CCTGTG	2	-	-	-	-	-	-	-	-	-	-	-	2	0.02
ACAGGG/CCCTGT	1	-	-	-	-	-	-	-	-	-	-	-	1	0.01
ACAGTG/ACTGTC	1	-	-	-	-	-	-	-	-	-	-	-	1	0.01
ACATGC/ATGTGC	1	-	-	-	-	-	-	-	-	-	-	-	1	0.01
ACCACG/CGTGGT	3	-	-	-	-	-	-	-	-	-	-	-	3	0.03
ACCACT/AGTGGT	1	-	-	-	-	-	-	-	-	-	-	-	1	0.01
ACCAGC/CTGGTG	2	-	-	-	-	-	-	-	-	-	-	-	2	0.02
ACCATC/ATGGTG	1	-	-	-	-	-	-	-	-	-	-	-	1	0.01
ACCATG/ATGGTC	2	-	-	-	-	-	-	-	-	-	-	-	2	0.02
ACCCAG/CTGGGT	1	-	-	-	-	-	-	-	-	-	-	-	1	0.01
ACCCCG/CGGGGT	1	-	-	-	-	-	-	-	-	-	-	-	1	0.01
ACCCGC/CGGGTG	4	-	-	-	-	-	-	-	-	-	-	-	4	0.05
ACCGCC/CGGTGG	4	-	-	-	-	-	-	-	-	-	-	-	4	0.05
ACCGGC/CCGGTG	2	-	-	-	-	-	-	-	-	-	-	-	2	0.02
ACCTCC/AGGTGG	1	-	-	-	-	-	-	-	-	-	-	-	1	0.01
ACGAGG/CCTCGT	2	-	-	-	-	-	-	-	-	-	-	-	2	0.02
ACGCAG/CGTCTG	1	-	-	-	-	-	-	-	-	-	-	-	1	0.01

ACGCCC/CGTGGG	2	-	-	-	-	-	-	-	-	-	-	-	2	0.02
ACGCCG/CGGCGT	5	-	-	-	-	-	-	-	-	-	-	-	5	0.06
ACGCGC/CGCGTG	1	-	-	-	-	-	-	-	-	-	-	-	1	0.01
ACGGAG/CCGTCT	1	-	-	-	-	-	-	-	-	-	-	-	1	0.01
ACGGCG/CCGTCG	4	-	-	-	-	-	-	-	-	-	-	-	4	0.05
ACGGGG/CCCCGT	3	-	-	-	-	-	-	-	-	-	-	-	3	0.03
ACTCCC/AGTGGG	2	-	-	-	-	-	-	-	-	-	-	-	2	0.02
ACTCCG/AGTCGG	1	-	-	-	-	-	-	-	-	-	-	-	1	0.01
ACTGCT/AGCAGT	2	-	-	-	-	-	-	-	-	-	-	-	2	0.02
ACTGGC/AGTGCC	3	-	-	-	-	-	-	-	-	-	-	-	3	0.03
ACTGGG/AGTCCC	3	-	-	-	-	-	-	-	-	-	-	-	3	0.03
AGAGCC/CTCTGG	1	-	-	-	-	-	-	-	-	-	-	-	1	0.01
AGAGGC/CCTCTG	1	-	-	-	-	-	-	-	-	-	-	-	1	0.01
AGAGGG/CCCTCT	5	-	-	-	-	-	-	-	-	-	-	-	5	0.06
AGATAT/ATATCT	1	-	-	-	-	-	-	-	-	-	-	-	1	0.01
AGATCC/ATCTGG	1	-	-	-	-	-	-	-	-	-	-	-	1	0.01
AGCAGG/CCTGCT	10	-	-	-	-	-	-	-	-	-	-	-	10	0.11
AGCATC/ATGCTG	1	-	-	-	-	-	-	-	-	-	-	-	1	0.01
AGCCCC/CTGGGG	2	-	-	-	-	-	-	-	-	-	-	-	2	0.02
AGCCGC/CGGCTG	1	-	-	-	-	-	-	-	-	-	-	-	1	0.01
AGCCGG/CCGGCT	4	-	-	-	-	-	-	-	-	-	-	-	4	0.05
AGCCTG/AGGCTC	2	-	-	-	-	-	-	-	-	-	-	-	2	0.02
AGCGGC/CCGCTG	2	-	-	-	-	-	-	-	-	-	-	-	2	0.02
AGCGGG/CCCGCT	4	-	-	-	-	-	-	-	-	-	-	-	4	0.05
AGGATG/ATCCTC	1	-	-	-	-	-	-	-	-	-	-	-	1	0.01
AGGCGC/CCTGCG	1	-	-	-	-	-	-	-	-	-	-	-	1	0.01
AGGCGG/CCGCCT	7	-	-	-	-	-	-	-	-	-	-	-	7	0.08
AGGGCG/CCCTCG	3	-	-	-	-	-	-	-	-	-	-	-	3	0.03
AGGGGC/CCCCTG	1	-	-	-	-	-	-	-	-	-	-	-	1	0.01
ATATCC/ATATGG	1	-	-	-	-	-	-	-	-	-	-	-	1	0.01
ATCCCC/ATGGGG	3	-	-	-	-	-	-	-	-	-	-	-	3	0.03
ATCCCG/ATCGGG	1	-	-	-	-	-	-	-	-	-	-	-	1	0.01
ATCGCC/ATGGCG	4	-	-	-	-	-	-	-	-	-	-	-	4	0.05
ATCGGC/ATGCCG	3	-	-	-	-	-	-	-	-	-	-	-	3	0.03
CCCCCG/CGGGGG	1	-	-	-	-	-	-	-	-	-	-	-	1	0.01
CCCCGG/CCGGGG	1	-	-	-	-	-	-	-	-	-	-	-	1	0.01
CCGCGG/CCGCGG	1	-	-	-	-	-	-	-	-	-	-	-	1	0.01
CCGGCG/CCGGCG	1	-	-	-	-	-	-	-	-	-	-	-	1	0.01

Supplementary Table S2: Analysis of 500 EST-SSRs.

Items	Number
Not amplify	62
Successfully amplified	438
Larger or smaller than expected size	69
Expected size	369
Polymorphic	112
Monomorphic	257
Total	500

Supplementary Table S3: *E. sibiricus* accessions used for marker validation.

No.	Source	Longitude (E)	Latitude (N)	Altitude (m)
Pop1	Lintan, Gansu	103°39'	34°56'	2336
Pop2	Lintan, Gansu	103°37'	34°45'	3010
Pop3	Luqv, Gansu	102°21'	34°29'	3481
Pop4	Luqv, Gansu	102°38'	34°33'	3032
Pop5	Luqv, Gansu	102°39'	34°30'	3023
Pop6	Luqv, Gansu	102°37'	34°05'	3389
Pop7	Hezuo, Gansu	102°54'	35°03'	2900
Pop8	Zhuoni, Gansu	103°34'	34°35'	2605
Pop9	Zhuoni, Gansu	103°36'	34°34'	2501
Pop10	Zhuoni, Gansu	103°01'	34°51'	3218
Pop11	Zhuoni, Gansu	103°37'	34°46'	2678
Pop12	Xiahe, Gansu	102°33'	34°45'	3171
Pop13	Xiahe, Gansu	102°49'	35°13'	2510
Pop14	Ruoergai, Sichuan	102°46'	34°07'	3126
Pop15	Maqv, Gansu	102°06'	33°59'	3475

Table S4. Characteristics of the 112 novel EST-SSR markers in *E. Sibiricus*.

Primer	Forward primer(5′-3′)	Reverse primer(5′-3′)	Tm (℃)	SSRs	Number of alleles	*Ho*	*He*	*PIC*	Transferability
ES-4	GGTAGACCCCCACGATCTC	AGTTGACTAGTGCTCATTCGCTC	60	CGC(3*5)	5	0.09	0.55	0.45	No
ES-5	CGTCTCCAAGGTGATGAAGTTT	ATCAGTGAAATGTGTCACACCC	60	GAT(3*5)	5	0.98	0.55	0.45	No
ES-7	CCTCCTCCGTTACCATGTTG	CCCTGCTTTTCCCTCTCTG	60	GCT(3*6)	3	0.91	0.54	0.43	Yes
ES-8	GTTCATCCTCCCCTTCAGATTC	GTACAAATCCCAGACCTGAGAAC	60	GCT(3*5)	4	0.05	0.55	0.45	Yes
ES-9	CTAACCACTGCAAGTCCCTCTC	AGTATCACCTGTTTGGTGTCGAG	60	CGA(3*6)	4	0.96	0.54	0.43	Yes
ES-10	CTAGCCACTGCAAGTCCCTCT	AGTATCACCTGTTTGGTGTCGAG	61	CGA(3*6)	4	1.00	0.54	0.43	Yes
ES-11	AGAACATCATCATCAAGAGGCAC	CTCCCCCTTCTCCTGCTC	60	GAG(3*5)	4	0.98	0.52	0.41	No
ES-16	GACGGGAAGCAGAAGCAG	GGAACGCGAAAATTAAAATAAGC	60	GAG(3*5)	5	0.04	0.56	0.46	No
ES-17	AGCGCTGACTGGTTTCTCTTTAG	GCATCAGGTTTTCTTCAGGTG	61	CGC(3*5)	3	0.05	0.52	0.41	No
ES-18	TCATCACTTCTTGCTTCTTTGGT	CATGCAGACACTACCTAGAGGGT	60	GCT(3*6)	5	0.09	0.55	0.45	Yes
ES-21	TGATGATGATTCAGCAAGTCAGT	AGGTTGGAAACTTAAAACCCAAA	60	TTC(3*6)	4	0.00	0.55	0.45	No
ES-22	AAGATATCCTGATGCTGGACAAA	GATCAGATCAATAGCTTGAGCG	60	CGGCT(5*4)	5	0.05	0.55	0.45	No
ES-23	CGTACTTGCGCCAGAAGTG	AGGTGTCCATCGAAGGGTC	60	CCG(3*5)	5	0.05	0.58	0.49	Yes
ES-24	AGTTGCTAGTTGTGCTTGTGTCA	CATCTGCGTACAAAACTGTGAAA	60	TG(2*8)	5	0.94	0.57	0.48	Yes
ES-25	AGCGATGATGTGAACAAGTAGGT	CTGACAAATACAGATGCACCAGA	60	TGC(3*6)	5	0.02	0.57	0.48	Yes
ES-40	TACTACCGAGGTATGTACAGGGG	GAAGAGGGCGAAGAAGGC	60	GGC(3*6)	4	0.05	0.57	0.47	Yes
ES-41	GTAAAACGTTGCCTGGTCCTC	CTCCAGCTGCAGAAACAGC	60	GGC(3*5)	4	0.04	0.54	0.43	Yes
ES-43	GACAAGGTTTGAGCGTTTTGAG	CTAGCAATCAATCGAGAGTCGTT	61	GCA(3*6)	5	0.04	0.58	0.49	Yes
ES-45	CTCCTCCCAGTACCTCTTCTGC	AACAACCATCCCCTGTGAACT	61	CGG(3*5)	5	0.09	0.56	0.46	No
ES-47	TACCTCCGAGAATGTTGCTCA	TCTTCTTTGAATTTACCAGCAGC	60	GGC(3*5)	4	1.00	0.58	0.49	Yes
ES-50	GTAGTAGAGCCAGTAGACGGCG	GACTGGTGGGTCTTCGACAC	61	GAC(3*5)	5	0.07	0.57	0.48	Yes
ES-51	GAGCTGAGCTGAGAAGAAAACAG	CACAATCATCTCATCTTCCTTCC	60	GA(2*10)	6	0.98	0.59	0.5	Yes
ES-59	AATCGTAGGAACAACAAAGGACC	GGCGGTAATACACCACCTTATTT	60	AG(2*6)	4	0.00	0.55	0.45	No
ES-64	TGTCCATATCAAATTCCAACTCC	CAAGGGCAAGAAGAAAACCAC	61	GTC(3*6)	7	0.05	0.58	0.49	No
ES-70	ACGCTGAACGGCTACCAG	CTACTCTGAGCTGCTTTACTCCG	60	GCA(3*7)	5	0.07	0.6	0.52	Yes
ES-71	TCCTGCTGTACATTTTGGTATCC	TGATGAGTCAAGACATGGAGCTA	60	GTG(3*5)	5	0.07	0.55	0.45	No
ES-72	GGAGAAGGCTGCTACCAAGA	CTTCTCCTCCTTCTTCTTTGGG	60	AGA(3*5)	5	0.07	0.55	0.45	Yes
ES-74	CCCACTGTTGTACCCTGAATTAT	GTGGTTCCTATGGTGGTGGT	59	CCA(3*5)	4	0.04	0.54	0.43	Yes
ES-75	ACCACCCTGGAGTAGCCATT	CTGGTCCATCGTCGACTTC	60	CTG(3*5)	4	1.00	0.54	0.44	Yes
ES-76	GCAGTAGAGCACCTTCACCG	AACCAAATCCGTCACGACTC	60	CCG(3*6)	4	0.09	0.56	0.46	Yes
ES-78	CGGTTCAGCATTAATCGAGTCT	ACATCTCAGCCAATGATCCTAGA	60	CT(2*6)	4	0.00	0.52	0.41	Yes
ES-82	ATTGACAAGCTCCTTGCAGAGAT	TTTTTCTTCTTGGATTTCTTCCC	61	GCT(3*5)	4	0.91	0.58	0.49	Yes
ES-86	CATTGTTACATTGCACAGCAGAT	CATGTTGAATGGTCCTCTTGG	60	GCC(3*5)	5	0.09	0.58	0.49	Yes
ES-91	AGTCGGTGACATACAAGCAATG	TTAATACCAGTAGCTTCCGCTTC	60	CGC(3*5)	5	0.09	0.58	0.49	Yes
ES-96	CGCAAGATCTTCATGTGGAC	GTTGAAGAACATGAGCGCCT	60	GCC(3*5)	3	0.98	0.51	0.39	No
ES-97	ACTGTGGGAGAAGGTGAGAGACT	CTTTCCTCCAGCTCATGGTG	61	GCG(3*5)	4	0.91	0.58	0.49	No
ES-100	AGAAGAGCAAGAGCCCAGG	ACCTCCTGATCATCTCCACCT	60	GAG(3*5)	3	0.05	0.52	0.41	No
ES-101	CTCTGTTACTTTGCAGCCTGTG	CACAAACCAACTGTGAACACATC	60	CA(2*6)	5	0.95	0.56	0.46	No
ES-103	GTTAGAGAAGGAGGCGGTATGG	CACTTCTCACTGTCCACTCCAAG	61	GCG(3*5)	4	0.00	0.54	0.43	Yes
ES-104	ATTCTACTGATGATTCCTCTCGG	TCCACCATATATACCCGACCATA	59	TGG(3*5)	4	0.00	0.54	0.44	No

ES-105	GGTGGAGAAGGGAGATGAGTC	AGGCTCATGAGGAACAAGTCTCT	60	GGA(3*5)	5	0.95	0.61	0.53	Yes
ES-106	GAAGAGCCGGATGGTGTC	ATAGCTAGGTATCTGTCTCGCCG	60	CTG(3*6)	4	0.04	0.54	0.43	No
ES-110	AGTCTCGATCTCGTAGAATCCCT	AGAGGAGGAAGAAGAGTGGGAA	60	CCT(3*6)	6	0.98	0.56	0.46	Yes
ES-113	CTCTCCGAGGGTGAGTGTCAT	GTGCCATCTCCAGCTCCTAC	61	CGC(3*6)	4	0.09	0.56	0.46	No
ES-116	GTGTTGTAGCAGTCGAGGAGG	GGTGGCTATGAGATGACCG	59	GCA(3*5)	6	0.14	0.74	0.7	No
ES-123	AGCATGAAGCTCGACTGTGAGT	GCGAGTACATCTCGTACTTCTGG	61	GAA(3*5)	5	0.96	0.56	0.46	Yes
ES-125	GAGCATCGACAGATTATTCCTTG	CGAAGGAACCTCTGCAAGAC	60	TG(2*9)	6	0.93	0.58	0.49	Yes
ES-131	TAGATTACCCCGTCTTCTTGAGG	GGAGAAGATCAACAACCTGCAC	61	CGG(3*6)	4	0.96	0.54	0.44	No
ES-133	GTTCAACAAGCCGAAATCAAG	CCGTCAAAGATATGAGGAATGAG	60	CAC(3*6)	5	0.93	0.58	0.49	No
ES-141	TTATGTGATTTCATAATTGGCCC	GCTGCTGCTACCGTTCTTATTTA	60	TTC(3*6)	6	0.98	0.58	0.49	Yes
ES-143	GCACGCCAGATCACCAGG	GTCGCGTAGTGGACCAGC	62	GCA(3*5)	5	0.20	0.58	0.49	No
ES-144	GGTAGTCGTTGACCCAGATGTC	CACATTGTAAACTGGTCCTCCTC	60	CGC(3*6)	4	0.05	0.54	0.44	No
ES-149	AGGAATTCAACCAAGAGGAGC	AAGAGCATGCTGGTGAGGATAC	60	AGA(3*5)	6	1.00	0.58	0.49	Yes
ES-155	CTCATCGCCTCCCACAAC	ACGGTTAGCACGGGACTAGAG	60	CAACGG(6*4)	6	0.05	0.58	0.49	No
ES-157	AATGGTGGAGAAGACGAAGAAG	GAACTTGTGGAGATGGAAGGTC	60	AGC(3*6)	8	0.02	0.83	0.81	No
ES-167	AACCTGCCCTTCTTCGAGAT	CCAAGATGCAGATAACCTCCAG	61	CTT(3*5)	5	0.98	0.55	0.45	No
ES-176	GTATTGGTCTCCTTAGCCTGGTC	ATGATTCCCAGGACAAAACTGAT	61	TGC(3*5)	6	0.21	0.66	0.6	Yes
ES-177	CTCGATGATCCCCAGCTC	GTCGTCGTCTTCGCCTTC	59	CAG(3*5)	8	0.96	0.58	0.49	No
ES-179	GCATGTCCTTTCCAAGAACTATAA A	TGCTCCAATCAAACAATTAATCA	60	TTC(3*5)	5	0.95	0.57	0.48	Yes
ES-180	TAGAGCAACTCTGGCATATCCTT	TGCTCCAATCAAACAATTAATCA	59	TTC(3*5)	5	0.98	0.54	0.43	Yes
ES-182	TCCTCCAGAATATTGAAGTCGTC	AAGACTCCATATGGTCCTTCCAT	60	CTG(3*5)	4	0.00	0.56	0.46	No
ES-192	GTGTACTGCGTGTCCTGTTGTT	CTACACGGACTCACGGGG	60	TGGGTC(6*4)	7	0.03	0.77	0.73	No
ES-193	CGGTTGTTCTGGTGGAGG	AGGTGAGCTTCGCGTACAAC	60	GTG(3*5)	4	1.00	0.58	0.49	Yes
ES-202	CATCAGCTCATCTGCGTATTTC	GAGAGATAACTCGCACAGCCAC	61	GGC(3*5)	6	0.95	0.6	0.52	No
ES-207	TTGTCTGAATGCACAGAGGTCTA	AAAAGAAGCAGACATGGTTTCAC	60	GAT(3*5)	6	0.05	0.58	0.49	Yes
ES-210	GCGTGTGCGTTTATATTTACCTC	GCCTCCGCTTATATATCGCC	60	AG(2*7)	5	0.98	0.56	0.46	No
ES-213	CTCGCACATCTCCACCTACTACT	GAAGTACCCGATCCTCCTCAC	60	GAG(3*5)	5	1.00	0.56	0.46	Yes
ES-214	TGGTGCAGCATGAACGAC	GCTCGTCTACCATCAGCTTCTT	60	TGG(3*5)	6	0.10	0.59	0.5	Yes
ES-217	CTCGGGCATCTTCACCAC	GATGAGAGGGTTGACGAAGAA	60	CCG(3*5)	3	0.10	0.54	0.44	Yes
ES-223	GTGACGGAGTAGAGCTTGACG	CACCTGATTCTTCTCCTTGCTG	61	CCG(3*5)	7	0.30	0.72	0.67	No
ES-226	GGATGGATGGATTTATACACGG	CTCCACTCCCAAATCTCATCTC	60	AGAA(4*5)	6	0.00	0.66	0.6	No
ES-229	AGCTCAAGTACAAGCTGTGGTTC	CTGCTGCTAGTATCCAGAGTGGT	60	AGC(3*5)	5	0.16	0.76	0.72	No
ES-230	GTCGGTGATGACGATAAGGTG	ATTCCTCTCCCTCTCTCCCTC	60	GGC(3*5)	4	0.07	0.69	0.63	No
ES-231	TAGCTGGTCATGCCTAGGAGTAG	CCAGGTGTCAGGATATAGCAAAA	60	GA(2*6)	6	0.93	0.58	0.49	No
ES-236	TCGCATGCTTATAATCCTTTGAC	TGAGGTCTCTGTCAATACCAACA	60	TTGCTC(6*4)	5	0.07	0.57	0.48	Yes
ES-239	TAGAGGCTGATTCCTCTTGAACC	AGGGAAACAGGCCAGACATAC	61	CCA(3*5)	5	0.98	0.56	0.46	No
ES-244	TTGGGCCCATACTCCTAACTATT	TCTGATGGTTGCAGATTTTTCTT	60	CA(2*6)	4	1.00	0.58	0.49	Yes
ES-253	CATCTCTTCAAACTTGGATTGGT	GTGATCTATACCATTGGCCTCAA	60	CT(2*8)	5	1.00	0.57	0.48	Yes
ES-257	AGGGAGGGGATGAGGAAAAT	GTCCTCATAGACTTCCCCTGTTC	61	ACG(3*7)	4	0.00	0.59	0.5	Yes
ES-259	CTCCTCTACCTGTCTGCTGCTA	AGATCGTCGACTACGTCAAGAAG	59	CGC(3*6)	4	0.05	0.67	0.61	Yes
ES-261	GCTGTTCTCATTGCTGTTGGTAT	ATTCACGTCAGTTGTTGGAGACT	60	GCT(3*6)	5	1.00	0.59	0.5	Yes
ES-275	GTACCAGCGCCCCATCTAC	GAGGAGGAGCACGACGAG	60	GCT(3*5)	6	0.10	0.61	0.53	No
ES-277	CCCCTCAGGTTGTTCATCAT	GGCTACACAGGCTCCTCCTAC	60	CTG(3*5)	6	0.09	0.68	0.62	No
ES-279	ATGAGGTTGAGGAGCAGGG	CGACCTTTTAACTTGCCAAGAG	60	CT(2*6)	3	0.09	0.54	0.43	Yes

ES-281	CACCTTGTACACGTCCTCCAC	ATTCCCATCTCGGTCAGCAT	61	CCG(3*6)	3	1.00	0.52	0.41	No
ES-282	GGAGCTTGACCTCCGTGTC	AAAACCCTAACCCTAGCACAGAC	60	CGC(3*5)	3	0.56	0.59	0.51	Yes
ES-303	GAGGAGGGGCTGCATTGT	GCAGTGACGCCTACATTCATT	61	GGT(3*5)	6	1.00	0.62	0.55	No
ES-309	TACCCGCAGTACGGGAACTACTA	AGGGTAGTGGTGGGAGTGG	61	CCG(3*5)	3	0.03	0.51	0.39	Yes
ES-310	CGTAGCAATTCCATTCTATCCAG	TGGTGAGCTAGATTGACACTGAG	60	GCT(3*5)	5	1.00	0.58	0.49	No
ES-316	ATCGTCTTCTCCATCTCCACC	GTGATTAATGGGGGAGGCAAC	62	GCC(3*5)	6	0.95	0.56	0.46	Yes
ES-320	GATTAAGTCGTCCAATTCCACC	CTGTAGTCGACTCTTCTCTTGGC	60	CAA(3*5)	5	0.24	0.7	0.65	Yes
ES-322	GGGTGTGATTCATAAAACGAATG	TCTTTCTCGTGACTGTTCCTTTC	60	AAT(3*5)	3	1.00	0.52	0.41	Yes
ES-335	GTAGCCGTAGAAGGAGAGGAGAG	CTACAGGAAACAACATCGCACTC	61	GAG(3*5)	4	0.98	0.52	0.41	No
ES-347	CATGAAGATGATGCGTGTTTTAAT	CCGACTCCTAATTGAACTCGTAA	60	TG(2*6)	5	0.96	0.58	0.49	Yes
ES-349	TAGGTGTGGGGAGGATATATGAG	TAAGCACCCTAATTAAAATGCCA	60	CT(2*9)	5	1.00	0.56	0.46	No
ES-350	GAGGAAGGAGGAGGAGAGAAAG	TCATCATACCCCTCTTCTTCTTG	60	CAA(3*6)	5	0.39	0.67	0.61	Yes
ES-352	CGTCTTCCGCTTCATCTTCTT	CAAAGATCCAGATCACACCAAAC	61	TCT(3*5)	6	0.93	0.61	0.53	Yes
ES-367	GGCGTCCTGATCTATACAAAGTG	TATTTATGCAAGGCGAAGCTAGA	60	CAT(3*5)	8	0.46	0.72	0.68	No
ES-374	ACGAACGATAAAAGAGACACAGG	GTCAGCTGAGGACCATCCC	60	GGT(3*5)	4	0.06	0.72	0.67	No
ES-379	CAAGGAGAGTCTTGCCTATACCA	GACACCATCTCAACTAGCAGACC	60	GCT(3*5)	5	0.86	0.58	0.49	No
ES-384	GTGATTTAGCTCGCAATTCTGAT	GATGAACAACAGAAGCAGATGGT	60	TGC(3*5)	8	0.85	0.73	0.69	No
ES-397	TGAGTTGATACGAGACTGGGATT	AAGGAGAGGAACGGAATCGTAT	60	GCT(3*7)	4	0.96	0.54	0.43	No
ES-399	ATTCTGGCTTTGTGGTGTCTTT	GATGCAACCATGTCTAGCTTTTT	60	GATGCT(6*4)	5	0.11	0.59	0.5	No
ES-401	CTAGGGTTACCCCTCCACGTC	ACGCGACGACAAATAACAAAG	61	CGG(3*5)	7	0.91	0.6	0.52	No
ES-405	AGAGAAAAGGAGATTCTCATCCC	GCTGCTCTGCATCCTACTCTATC	59	CCCTG(5*4)	7	0.02	0.59	0.51	No
ES-408	CCACAAATGTTGTCATGAACTGT	TGTGTCTCAAGCTTACGAACTGA	60	AGG(3*5)	5	0.76	0.61	0.54	No
ES-429	TTCTGGTGAGGATGAGGTTATGT	CTCTCGTCTTCGCTGTCTGATT	60	GAC(3*5)	9	0.88	0.67	0.62	No
ES-457	CTTGGAGTATATCCTGGCCGT	GACGAGGAGGAAGAGGATGAG	60	CGT(3*5)	8	0.79	0.66	0.6	No
ES-467	GAGGAAGAGTGATGAGGATGATG	CTGGTTCTCCCTTGTCTTTCTTT	60	AGA(3*5)	3	0.45	0.62	0.55	Yes
ES-479	GAAAGTGCCTCTTGTGAGAAATG	GGAAGAATAACAAGGTGTCACCAG	61	CTT(3*5)	5	1.00	0.63	0.56	No
ES-493	GACTTCTCGGTGTCGTCGTC	GCTCCCCTACGAGCTGTTC	60	CTT(3*5)	4	0.79	0.58	0.5	No
ES-494	GGTCTTTGCACCACAGAATGTA	CTTCCTCTCGTCCAGTCTCTTCT	60	CTT(3*5)	4	0.09	0.54	0.44	Yes
Mean ± sd	-	-	-	-	4.94 ±1.25	0.49 ±0.4	0.59 ±0.6	0.50 ±0.8	-

Table S5. Average of the polymorphism information of 55 EST-SSR markers within 14 different *Elymus* species.

No.	Species	Number of alleles	*Ho*	*He*	*PIC*
1	*E. abolinii*[a]	1.65	0.52	0.30	0.23
2	*E. antiquus*[a]	1.85	0.53	0.32	0.26
3	*E. burchan-buddae*[a]	2.38	0.59	0.45	0.38
4	*E. caninus*[a]	1.62	0.35	0.26	0.21
5	*E. ciliaris*[a]	2.06	0.64	0.40	0.33
6	*E. gmelinii*[a]	2.13	0.55	0.40	0.33
7	*E. longearistatus*[a]	2.20	0.44	0.41	0.34
8	*E. nevskii*[a]	2.91	0.59	0.53	0.47
9	*E. panormitanus*[a]	2.78	0.39	0.53	0.46
10	*E. semicostatus*[a]	2.02	0.53	0.34	0.29
11	*E. tschimganicus*[a]	1.98	0.62	0.37	0.31
12	*E. barbicallus*[a]	2.46	0.75	0.51	0.43
13	*E. macrochaetus*[a]	2.05	0.75	0.43	0.35
14	*E. sibiricus*[b]	4.62	0.50	0.57	0.48
	Mean ± sd[c]	2.16±0.39	0.56±0.12	0.40±0.09	0.34±0.08

Note: 'a' denotes this species contains three individual plants; 'b' denotes this species contains 45 individual plants; 'c' denotes the mean values of No. 1 to 13 *Elymus* species, and not included *E. sibiricus*; 'Number of alleles', '*Ho*', '*He*', and '*PIC*' represent the mean values of 55 EST-SSR markers in each species.

Table S6. Plant materials used for cross-species transferability. Accession No. is provided by The U.S. National Plant Germplasm System (NPGS).

No.	Latin Name	Genome	No. of individuals	Accession No.	Source
CK	*Elymus sibiricus* L.	StStHH	Pop3	-	Luqv, Gansu
			Pop12	-	Xiahe, Gansu
1	*E. abolinii*	StY	1	PI 499585	China
			2	PI 499585	China
			3	PI 499585	China
2	*E. antiquus*	StY	1	PI 564957	Xizang, China
			2	PI 564957	Xizang, China
			3	PI 619528	Sichuan, China
3	*E. burchan-buddae*	StY	1	PI 636649	Gansu, China
			2	PI 655143	Sichuan, China
			3	PI 655210	Xizang, China
4	*E. caninus*	StH	1	PI 639752	Xinjiang, China
			2	PI 639752	Xinjiang, China
			3	PI 639752	Xinjiang, China
5	*E. ciliaris*	StY	1	PI 531575	China
			2	PI 531575	China
			3	PI 564916	Russian Federation
6	*E. gmelinii*	StY	1	PI 639761	Russian Federation
			2	PI 655100	Xinjiang, China
			3	W6 21543	Mongolia
7	*E. longearistatus*	StY	1	PI 401275	Iran
			2	PI 401281	Iran
			3	PI 401282	Iran
8	*E. nevskii*	StY	1	PI 564925	Russian Federation
			2	PI 632570	Mongolia
			3	PI 632570	Mongolia
9	*E. panormitanus*	StY	1	PI 254866	Iraq
			2	PI 254866	Iraq
			3	PI 561099	Bitlis, Turkey
10	*E. semicostatus*	StY	1	PI 271522	Himachal Pradesh, India
			2	PI 564964	Pakistan
			3	PI 564964	Pakistan
11	*E. tschimganicus*	StStY	1	PI 499481	China
			2	PI 547371	Kyrgyzstan
			3	PI 547371	Kyrgyzstan
12	*E. barbicallus*	StY	1	PI 504441	China
			2	PI 504441	China
			3	PI 504441	China
13	*E. macrochaetus*	StY	1	PI 564944	Kazakhstan
			2	PI 564945	Kazakhstan
			3	PI 564945	Kazakhstan
